# The effect of interface heterogeneity on zinc metal anode cyclability†

**DOI:** 10.1039/d4ta03165b

**Published:** 2024-08-14

**Authors:** J. T. Simon, V. Šedajová, D. Tripathy, H. E. Smith, S. M. Clarke, C. P. Grey, S. Menkin

**Affiliations:** a Yusuf Hamied Department of Chemistry, University of Cambridge Lensfield Road Cambridge CB2 1EW UK sm2383@cam.ac.uk; b Institute for Energy and Environmental Flows, University of Cambridge Madingley Road Cambridge CB3 0EZ UK; c The Faraday Institution, Quad One Harwell Science and Innovation Campus, Didcot OX11 0RA UK

## Abstract

Zinc metal batteries (ZMBs) are promising candidates for low-cost, intrinsically safe, and environmentally friendly energy storage systems. However, the anode is plagued with problems such as the parasitic hydrogen evolution reaction, surface passivation, corrosion, and a rough metal electrode morphology that is prone to short circuits. One strategy to overcome these issues is understanding surface processes to facilitate more homogeneous electrodeposition of zinc by guiding the alignment of electrodeposited zinc. Using Scanning Electrochemical Microscopy (SECM), the charge transport rate on zinc metal anodes was mapped, demonstrating that manipulating electrolyte concentration can influence zinc electrodeposition and solid electrolyte interphase (SEI) formation in ZMBs. Using XPS and Raman spectroscopy, it is demonstrated that an SEI is formed on zinc electrodes at neutral pH, composed primarily of a Zn_4_(OH)_6_SO_4_·*x*H_2_O species, its formation being attributed to local pH increases at the interface. This work shows that more extended high-rate cycling can be achieved using a 1 M ZnSO_4_ electrolyte and that these systems have a reduced tendency for soft shorts. The improved cyclability in 1 M ZnSO_4_ was attributed to a more homogeneous and conductive interface formed, rather than the bulk electrolyte properties. This experimental methodology for studying metal battery electrodes is transferable to lithium metal and anode-free batteries, and other sustainable battery chemistries such as sodium, magnesium, and calcium.

## Abstract 2

The surface of a zinc metal anode was mapped using SECM. Slow Zn^2+^ transport can be caused by an insulating SEI (ZHS or ZnO) or horizontally plated zinc. Fast Zn^2+^ transport can be caused by a protruding morphology or a more conducting SEI.

## Graphical abstract

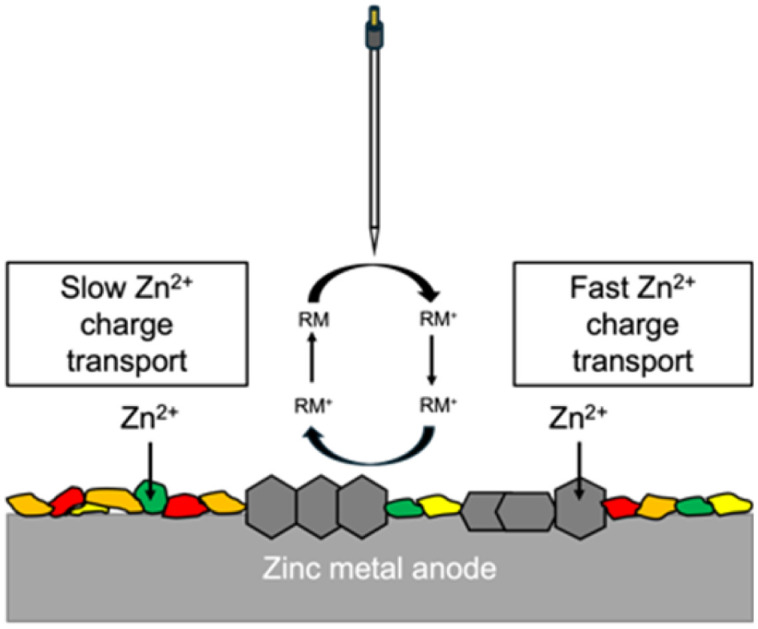

## Introduction

In the face of the looming climate catastrophe, there is a need to diversify the types of batteries that are used around the globe to aid sustainable energy storage across multiple sectors. Lithium-based technologies are currently the forerunners for mobile energy storage applications, attributed to their high energy density, but have concerns regarding their safety, sustainability, and raw materials mining. Hence, other more intrinsically safe and sustainable systems are desirable, particularly for large-scale grid storage applications where weight is not a primary factor, and the abundance and recyclability of the components are more critical.

Zinc metal batteries (ZMBs) are lower-cost than lithium-ion batteries, safer, and a more environmentally friendly alternative system to their lithium counterparts. They combine a high theoretical capacity (820 mA h g^−1^) with a low electrochemical potential (−0.76 V *vs.* SHE), making ZMBs a promising candidate for grid storage applications.^1–5^ Another significant advantage of ZMBs is that the most commonly used salt in the electrolyte, ZnSO_4_, is not fluorinated and can be used as an electrolyte under close to neutral pH, therefore making it significantly safer.^6,7^

Rechargeable ZMBs operate using repeatable and reversible electroplating and electro-stripping of zinc onto a zinc metal anode. Unfortunately, these processes are usually accompanied by several issues: the competing hydrogen evolution reaction (HER, which occurs at approximately the same potential as zinc electroplating), surface passivation and excessive interphase growth, corrosion, a protruding morphology that encourages active material loss due to the creation of zinc metal shards that break away from the electrode (forming “dead zinc”), and short circuit formation.^8,9^ These mechanisms are all dominated by surface reactions and contribute to low zinc plating-stripping efficiency and capacity losses. Deposits of zinc with high local curvature that form due to inhomogeneous nucleation elevate the local electric fields and local current densities, which in turn leads to the strong adsorption and then reduction of Zn^2+^ further aggravating the growth of these undesirable protrusions.^10^

One strategy to aid the understanding of how zinc electroplates is to determine whether, and how, a solid electrolyte interphase (SEI) forms in these systems. The most commonly suggested SEI formation mechanism on a zinc metal anode differs from that on an alkali metal anode.^11–13^ In a zinc system, the SEI is reported to form *via* a local pH increase during the hydrogen evolution (2H_2_O + 2e^−^ → 2OH^−^ + H_2_) that accompanies electroplating (Zn^2+^ + 2e^−^ → Zn^0^), rather than *via* electrolyte reduction. The freshly electroplated zinc is in a more reactive state, which further aggravates the HER. The resulting pH change gives rise to the precipitation of ZnO, which has been shown to be present as a major component of the SEI.^14^ Jäckle *et al.* attributed the dendritic zinc morphology to ZnO layer formation instead to an inherent zinc metal property.^15^ Other studies have reported the formation of SEI compounds such as Zn_4_(OH)_6_SO_4_·*x*H_2_O (ZHS) in the presence of SO_4_^2−^.^5^

The crystallinity of the metal deposits has a major effect on zinc morphology due to the extraordinarily high anisotropy associated with zinc crystal structure. A crystal tends to maximise the exposure of its lowest-energy facet, here the {0 0 2} basal planes, resulting in platelets formed from two {0 0 2} planes. The crystal is constrained by vertical {1 0 1} facets on the sides, determining the hexagonal “Wulff shape” of zinc. However, Wulff shapes are built assuming the crystal is in a vacuum. In solution, an interphase that adheres to a specific facet can dominate the stable shape of the grown crystal. For example, Xin *et al.* have demonstrated that when ZHS serves as an SEI it suppresses undesirable dendrite growth by guiding the horizontal alignment of electrodeposited zinc flakes.^16^ The alternative perpendicular orientation is an undesirable dendritic form of growth. Without proper regulation, the newly deposited zinc flakes randomly orient to form moss-like zinc dendrites, leading to reduced coulombic efficiency. Subsequent inefficient stripping of the zinc close to the electrode surface and corrosion mechanisms can lead to “dead zinc”, which forms due to stripping and corrosion mechanisms.^17^ The resultant heterogeneous surface encourages further dendrite growth, and eventually battery failure. However, research on the zinc interphase (Zn-SEI) is scarce, and the properties of Zn-SEIs and their homogeneity still need to be explored.

Electroplating requires that the thermodynamic potential is strong enough to drive zinc reduction. However, additional energy is required to overcome other barriers to deposition, and this additional electromotive force is called the overpotential (*η*_t_) (eqn (1)). The overpotential developed during plating and stripping with a non-SEI forming metal (*e.g.* copper) originates from the ohmic resistance (*η*_IR_) of the system, which is mainly attributed to current collectors, electrolyte conductivity, and charge-transfer resistance (*η*_ac_) at the electrode surfaces. The charge-transfer resistance can also include a contribution from an overpotential required to overcome the activation barrier of metal nucleation. Any concentration polarisation (*η*_c_), established during plating due to metal ion depletion near the plated electrode, also adds to *η*_t_.^18,19^

An SEI is also expected to introduce an additional overpotential (*η*_SEI_) due to hindered metal-ion transport through the interphase layer and potentially ion concentration polarisation at the SEI-electrolyte or SEI-electrode interface. Hence, the overall overpotential in these systems can be summarised as:

1 *η*_t_ = *η*_IR_ + *η*_SEI_ + *η*_ac_

Prolonged galvanostatic cycling of symmetric cells has been regarded as a key metric indicating the viability of a particular metal anode-electrolyte system. The voltage traces – *i.e.*, changes in overpotential, during plating and stripping yield information on the nucleation and growth of metal microstructures. Prolonged cycling can, however, lead to short circuits, where direct contact between the electrodes can occur through the deposited metal, creating an abnormal electrical circuit that allows current to travel along an unintended path with very low resistance.

Most research efforts to date to address the challenges of using zinc anodes focus on controlling the parasitic reactions *via* controlling electrolyte solvation structure and zinc surface pre-treatment (typically using expensive and hazardous fluorinated salts and organic co-solvents). The concentration of ZnSO_4_ aqueous electrolytes determines electrolyte solvation structure.^20,21^ ZnSO_4_, when dissolved in water, forms a solvent separated ion pair (SSIP) (or outer sphere complex), [Zn(OH_2_)_6_]SO_4_ and contact ion pair (CIP), [Zn(OH_2_)_5_OSO_3_] (inner sphere complex). The first solvation shell of Zn^2+^ contains six water molecules to form [Zn(H_2_O)_6_]^2+^ due to strong electrostatic interactions, and the second shell contains nine water molecules. The anion SO_4_^2−^ is only present in the second shell. The proportion of CIP increases with ZnSO_4_ concentration.^22,23^ Thus, when ZnSO_4_ is more concentrated, the higher concentration of SO_4_^2−^ results in fewer water molecules in the zinc ion solvation shell, which in turn lowers de-solvation energy and overpotential. As a result, the extent of zinc corrosion and hydrogen evolution decreases, and cycling efficiency increases.^24^

In addition to electrolyte optimization, whereby the solvation of Zn^2+^ is controlled to alter the desolvation energies and aid homogeneous deposition of zinc metal, there are a variety of other strategies used to contribute to understanding how zinc electroplates.^25^ The separator also plays a role in the transport of Zn^2+^ towards the anode surface, and modification of separator structure and properties can help facilitate the deposition of zinc metal and minimise parasitic side reactions.^26,27^ Alternatively, the structure of the electrode can be modified to make them more zincophilic to facilitate more uniform deposition, or surface coatings such as polymers can be added to the electrode to homogenise the electric field distribution and control the morphology of plated zinc.^28–30^

Scanning Electrochemical Microscopy (SECM), an extremely powerful technique for investigating local reactivity and topography, and which is starting to emerge as a method to study zinc batteries. For example, SECM was previously used by Zhao *et al.* to investigate the effect of the native oxide and its reaction with the electrolyte on a single plating of zinc in *ex situ* studies of plated electrodes.^31^ Here we move beyond this work by exploring interface heterogeneity during cycling. Specifically, by electrochemically imaging the interface at different stages of cycling, an *in situ* methodology for investigating interface homogeneity and the development of an interphase is presented. Electrochemical activity and surface information is combined, the results demonstrating that SECM is a powerful tool for investigating local reactions for battery electrode study. SECM was used to track the properties of the metal–electrolyte interface dynamic interface during different stages of zinc plating and stripping.

This work aims to understand the inherent formation mechanism of the SEI during zinc plating and stripping in ZnSO_4_ electrolyte solutions and use the insights to direct zinc plating and stripping towards more efficient and sustainable ZMBs. Given the limited applications of this method to the Zn-battery community we start by outlining the experimental SECM methodology. The interface is electrochemically formed and then imaged at different stages of cycling, and an *in situ* methodology for investigating interface heterogeneity and growth of an interphase was developed. By combining electrochemical activity and surface information, it is shown that SECM is a powerful tool for investigating local reactions for battery electrode study. SECM is combined with more traditional electrochemical methods, XPS and Raman spectroscopy, to show that the SEI-like layer on zinc (denoted Zn-SEI) starts forming during the first plating, however, it only achieves full coverage on the anode after ten cycles. More broadly, this work demonstrates the critical role of surface reactions in determining the cyclability of zinc metal anodes.

## Methodology: tracking zinc interface evolution using scanning electrochemical microscopy

**Fig. 1:**
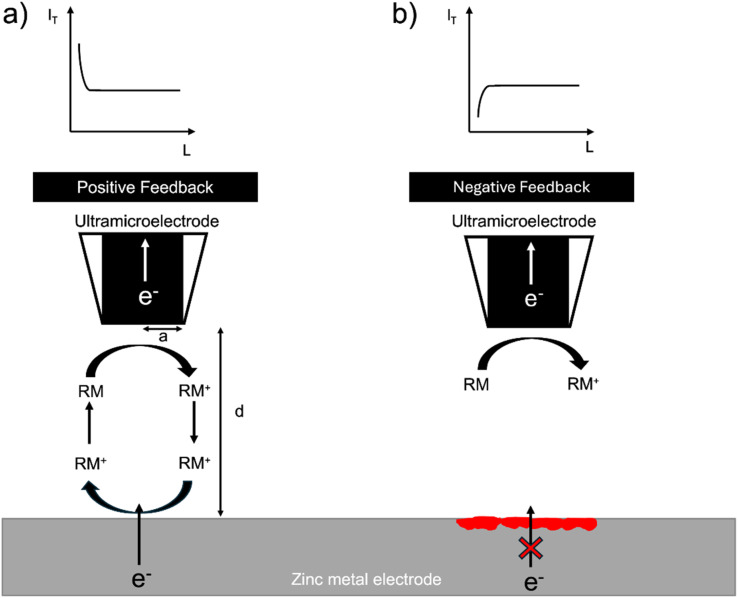
(a) A metal provides positive feedback because electrons can be provided to the redox mediator from the electron-conducting substrate. Positive feedback is shown as an increase in tip current (*I*_T_) with a decrease in distance from the surface (*d*). The distance from the surface (*d*) is normalised with the UME radius (*a*) and labelled (*L*), *i.e.*, *L* = *d*/*a*. (b) When an insulating electrode is probed, the electrons cannot tunnel through it and cannot be provided to the redox mediator and its diffusion to the UME is hindered; hence, negative feedback is observed. Negative feedback is shown as a decrease in tip current (*I*_T_) with a reduction in distance from the surface (*d*) due to the limited diffusion of the redox shuttle near the surface. An SEI or oxide layer (depicted as a red coloured layer on the metal electrode) could block the electrons tunnelling from the metal and result in negative feedback, similarly to the feedback from an insulating surface. If this layer does not fully cover the metal, the response will be neither ideally positive nor negative.

The SECM setup uses the electrochemical response of a redox mediator to map local electron transfer and thereby map the electrochemical heterogeneity of the interface. The local feedback response is controlled by both the electronic conductivity and the topology of the interface and is recorded as the normalised tip current over a given region (*I* = *I*_T_/*I*_T,∞_), where *I*_T,∞_, is the steady state diffusion limited current (*i.e.* far from the surface) and, *I*_T,_ the tip current at a location close to the surface. In general, when the surface is conducting (*I* > 1), positive feedback is recorded, and when the surface is insulating (*I* < 1), negative feedback is recorded. The normalised tip current (*I*) is plotted *vs.* the normalised distance (*L* = *d*/*a*), where *a* is the radius of the ultramicroelectrode (UME) and *d* is the distance of the UME from the surface. The resulting plot is called a probe approach curve (PAC) (Fig. 1).^32^

Zinc metal can provide positive feedback because electrons can be provided to the redox mediator from the metal. Positive feedback is shown as an increase in tip current (*I*_T_) with a decrease in distance from the surface (*d*). However, if an insulating oxide is present or an interphase grows to a thickness such that electrons cannot tunnel through it, electrons cannot be provided to the redox mediator and negative feedback is observed. Negative feedback is shown as a decrease in tip current (*I*_T_) with a decrease in distance from the surface (*d*) due to the hindered diffusion of the mediator to the tip (Fig. 1).

In this system, the feedback response is controlled by the morphology of the electroplated zinc and the electronic resistance of the surface. If the surface is covered by an electrically insulating interphase (SEI), the feedback response is expected to be negative. The orientation of the plated zinc also determines the type of feedback observed. If the plated zinc is horizontally aligned and passivated (*e.g.* formation of ZnO), the response is negative, as shown in a recent SECM study by Zhao *et al.*^31^ However, if the plated zinc is vertically aligned, the feedback response will be positive.^31^ Positive feedback can also be recorded due to an increase in the height of the morphology.

## Results

**Fig. 2:**
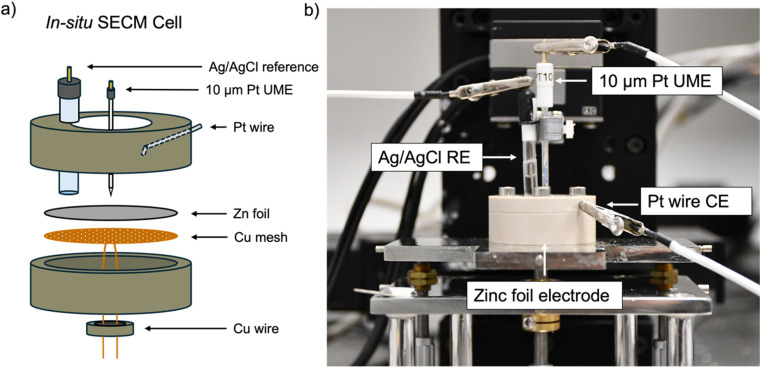
(a) Schematic of the *in situ* 4-electrode scanning electrochemical microscope cell using a zinc foil working electrode, a 10 μm platinum ultramicroelectrode, a Pt wire counter electrode and a saturated Ag/AgCl reference electrode. (b) A photo of the *in situ* scanning electrochemical microscopy cell used with the CH Instruments 920C SECM instrument.

All experiments used an *in situ* SECM cell that was developed in house (Fig. 2). A zinc foil electrode was used as a substrate electrode and electrically connected to a potentiostat using a Cu-mesh current collector in a 4-electrode configuration. 1 mM ferrocenemethanol (FcMeOH) in 0.01 M ZnSO_4_ was used as the redox mediator *via* the FcMeOH/FcMeOH^+^ redox couple (Fig. S1† for details on the choice of the redox mediator). The interface was tracked by SECM, while plating and stripping were performed by plating and stripping a total of 1.7 mA h cm^−2^ (equivalent to 4 mg cm^−2^ of zinc plated) per cycle at 1.7 mA cm^−2^ using either 2 M or 1 M ZnSO_4_ electrolyte. The same region of the zinc-metal electrode was electrochemically imaged using SECM at different stages of cycling.

### Probe approach curves

**Fig. 3:**
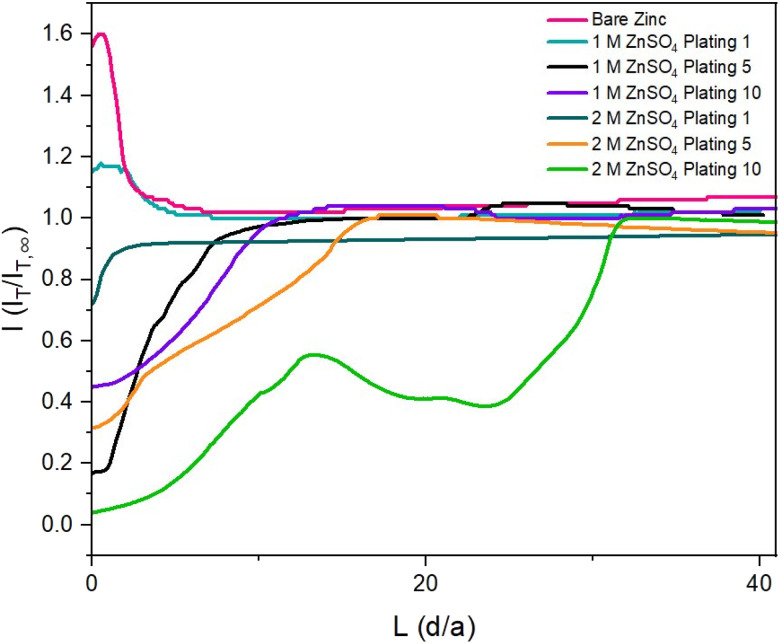
Probe approach curves measured at various stages of cycling at 1.7 mA cm^−2^. Measurements were recorded after the first, fifth, and tenth plating for zinc-metal anodes in 1 M and 2 M ZnSO_4_. An increase in tip current beyond *I* = 1 denotes positive feedback, and a decrease in tip current below *I* = 1 denotes negative feedback. A charge of 1.7 mA h cm^−2^ (4 mg cm^−2^ zinc) was passed per plating/stripping cycle.

To observe how the interface evolves during cycling, probe approach curve (PAC) measurements and SECM images were first taken at single points on the surface. Initially, the bare zinc surface, as received before cycling, was characterised. The PAC measurements were then repeated after the first, fifth, and tenth plating. These measurements were recorded for both 2 M and 1 M ZnSO_4_ electrolytes. The results are plotted as normalised tip current (*I* = *I*_T_/*I*_T,∞_) against normalised distance. Probe approach curves were measured by moving the UME towards the zinc-metal electrode at 1 μm s^−1^ and were stopped when the electrode was at the surface (*L* = 0 is defined as the point of contact of the UME with the surface). This is evident when the shape of the normalised tip current changes abruptly due to the bending of the electrode, according to the standard methodology of this technique (Fig. 3).

The first feedback measurement taken was bare zinc, as received (there was no potential bias on the substrate) before it was used in 1 M and 2 M ZnSO_4_ electrolytes. The data in Fig. 3 (pink line) shows a rising current on approach, indicating positive feedback, as expected for electron transfer to FcMeOH from the conducting zinc surface. This indicates that the pristine, as received zinc surface is not blocked by a significant passivation layer or an electrically insulating interphase prior to cycling. The maximum current was 1.6 times the steady state current (*I*_T,∞_, current recorded in the bulk, *i.e.*, at an infinite distance or ten times the tip radius from the surface).

#### 1 M ZnSO_4_ electrolyte

The zinc electrode PAC measurements in 1 M ZnSO_4_ after one plating also showed positive feedback (Fig. 3, turquoise line). This indicates that, in the region that was approached, an electrically insulating interphase has not yet accumulated to an extent enough to block electron flow. However, the positive feedback response is less than that for the bare zinc electrode surface, indicating that the rate of electron transfer of the metal to the redox mediator has decreased. Alternatively, this behaviour could be attributed to the zinc being plated with vertical alignment, which may not passivate as easily as horizontally aligned zinc, resulting in positive feedback.^31^ The PACs recorded after the fifth and tenth plating demonstrate negative feedback, where the current decreases relative to the bulk value (Fig. 3, black and purple lines). This is attributed to the accumulation of electrically insulating interphases such as ZnO and Zn(OH)_2_ or ZHS (associated with hydrogen evolution and local pH changes).^16^ Thus, this suggests that the SEI accumulates between cycles 1 and 5 for the 1 M ZnSO_4_ electrolyte system. This conclusion was further justified by measuring probe approach curves after each plating until the fifth plating (Fig. S2†). The measurements show that although positive feedback is shown after plating 1, negative feedback is measured for all subsequent plating cycles. This result is consistent with our findings depicted in Fig. 3.

#### 2 M ZnSO_4_ electrolyte

PAC data for the 2 M ZnSO_4_ electrolyte shows negative feedback after the first, fifth, and tenth plating (Fig. 3, teal, orange, and green lines). This indicates that an electrically insulating interphase accumulates more quickly for a 2 M than a 1 M system. This may be due to both bulk or surface effects. For example, for the same rate of production of OH^−^, higher concentrations of Zn^2+^ and SO_4_^2−^ would be expected to precipitate ZHS more quickly. Further, there could be a change in the solvation structure of the Zn^2+^ in a more concentrated electrolyte. Alternatively, plating more horizontally aligned zinc, which passivates more quickly, would also lead to negative feedback.^31^

The shape of the PAC deviates from the ideal negative feedback (Fig. 1) when the zinc electrode is scanned after the fifth and tenth plating (Fig. 3, orange and green lines) in 2 M ZnSO_4_ and after the tenth plating (Fig. 3, purple line) in 1 M ZnSO_4_. Specifically, instead of the typical exponential decay, the relative current linearly decays, with occasional decay rate changes and sometimes increases in relative current (Fig. 3, green line). This behaviour could result from the current measured *via* the UME, which travels vertically through the deposited materials, besides zinc shards, and senses regions of electron-conducting and non-conducting electrode surfaces. However, repeat data demonstrates that the trend of deviating from pure negative feedback is not always observed (Fig. S3†). Since the size of the ‘dendrites’ is of the same order of magnitude as the UME, similar responses are expected on approaching a dendrite and the surface. The shape of the curve is dependent on the local environment; however, all PACs (Fig. 3, orange, green and purple and Fig. S3†) show an abrupt current decrease further from the surface than a typical negative PAC (such as the one measured after the first plating in 2 M ZnSO_4_); hence, in these cases, the shape of the PAC was affected by the rough electrode morphology. The PAC after the fifth plating in 1 M ZnSO_4_ (Fig. 3, black line) shows a typical negative feedback response, however, the exponential decay takes place further from the surface than the other approach curves, potentially due to relatively homogeneous vertical growth of the surface.

### SECM images

The PAC measurements at a single surface location only provide information about a very small region of the surface. Thus, to explore how the tip current changes and to investigate the dynamic electrochemical activity of the surface over a larger area, SECM images were taken following the PAC measurements after the first, fifth, and tenth plating (see details on the plating process and the plating curves in the ESI Fig. S4–S23†). The images were recorded over a 300 × 300 μm area at a constant height of 10 μm above the surface.

SEM images were also taken of cycled electrodes to aid the physical interpretation of the SECM images. Note that the view field of the SEM is much smaller compared to the SECM images. SEM images with a larger view field, which capture a larger area of the surface, can be found in Fig. S46–S53.†

#### 2 M ZnSO_4_

**Fig. 4:**
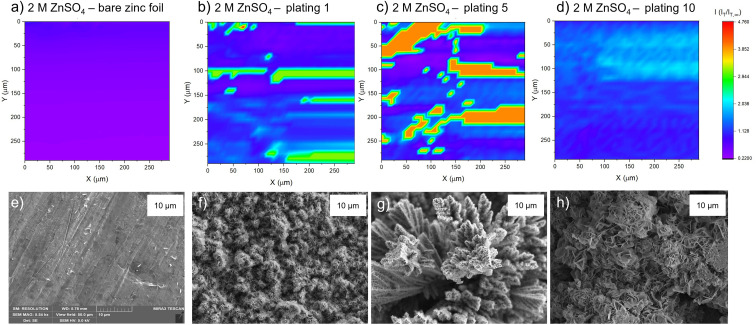
Scanning electrochemical microscope images of the same region of a zinc metal anode upon different stages of cycling at 1.7 mA cm^−2^ in 2 M ZnSO_4_. The colour scale represents the normalised tip current and is consistent across all the images. (a) Bare zinc electrode. (b) After the first plating. (c) After the fifth plating. (d) After the tenth plating. SEM image of (e) zinc electrode soaked in electrolyte for one hour, (f) after the first plating, (g) after the fifth plating, (h) after the tenth plating. SEM images were taken with an excitation voltage of 5 kV in resolution mode with a view field of 50 μm.

The SECM map of the zinc electrode, prior to any cycling (‘as received’; Fig. 4a), shows an essentially homogeneous surface, with negative feedback demonstrated across the imaged region, illustrated by a deep purple/pink colour across the entire map. This observation is different to that measured in the PAC above (Fig. 3, pink line), where positive feedback was seen, indicating an electrically conducting surface. It is likely that in the time before the experiment, the surface was oxidised to form an insulating ZnO layer, leading to the negative feedback. This is similar to that seen by Zhao *et al.*,^31^ who mapped bare zinc samples immediately after soaking.

The corresponding SEM shows a zinc electrode that was soaked in 2 M ZnSO_4_ for an hour prior to SEM imaging (Fig. 4e). The image shows surface roughness typical of a metal film, but with no large protrusions or variations in surface topology, consistent with the SECM image where no significant variations in the current response was observed across the image.

After the first plating, the surface becomes more heterogeneous, signified by the changing tip current across the map and higher current patches evident in a transition from purple to blue (Fig. 4b). This signifies that the surface has become more conductive after the first plating. The extremely well-defined areas of high current (shown in green) are attributed to protruding metallic features in this area. This is further evidenced by the corresponding SEM image (Fig. 4f), which shows the appearance of small agglomerations of zinc shards/flakes protruding from the surface. These agglomerations introduce a dimension of spatial heterogeneity to the SECM, as the image shows that the surface is no longer at a constant height, and some features protrude more than others. This is likely what is being recorded as regions of higher current in the SECM image.

After the fifth plating (Fig. 4c), the image becomes even more heterogenous, with more regions of negative feedback in purple. More prominently, the features that were identified in the SECM image after the first plating (Fig. 4b) have increased in size, and the tip current recorded above these features has also increased from approximately two times the bulk steady state current (measured by the UME in the electrolyte bulk) to four times the steady state current. These images suggest that between the first and fifth cycle, plated metal grows on previously protruding features, attributed to the tip effect.^33^ The SEM image (Fig. 4g) shows regions of very large dendritic growths protruding on the surface, which would explain the increased UME current due to an increase in the substrate height.

Interestingly, the SECM image taken after the tenth plating (Fig. 4d) shows a decrease in normalised current across the entire image compared to the fifth plating. The signatures of the plated metal can still be seen as regions of slightly higher current on the surface (lighter blue regions). Furthermore, the region seems more homogeneous than the fifth plating, with a lower current range across the entire image. The SEM image (Fig. 4h) shows larger agglomerated regions of zinc on the surface of the electrode. The agglomeration of small deposits into larger deposits leads to a more homogeneous interface, as probed by SECM. The lower current detected could be indicative of a more electrically insulating interphase accumulating on the surface.

#### 1 M ZnSO_4_

**Fig. 5:**
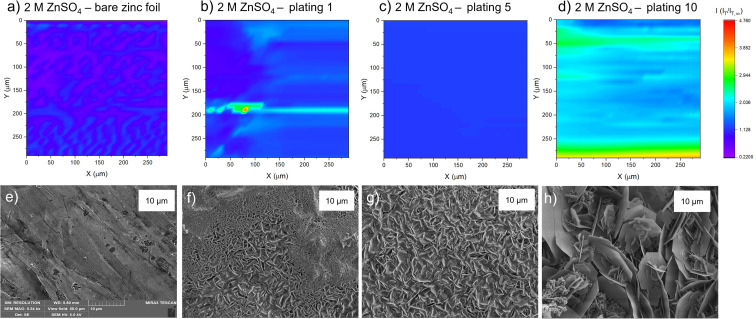
Scanning electrochemical microscope images of the same region of a zinc metal anode upon different stages of cycling at 1.7 mA h cm^−2^ in 1 M ZnSO_4_. (a) Bare zinc electrode. (b) After first plating. (c) After the fifth plating. (d) After the tenth plating. SEM image of (e) zinc electrode soaked in electrolyte for one hour, (f) after the first plating, (g) after the fifth plating, (h) after the tenth plating. SEM images were taken with an excitation voltage of 5 kV in resolution mode with a view field of 50 μm.

The SECM image for the zinc electrode prior to any electrochemistry recorded for the 1 M ZnSO_4_ electrolyte shows a slight heterogeneity in the low current range (Fig. 5a). Interestingly, the variations in normalised tip current match the orientation of the morphology/striations on the bare zinc metal surface, which can be seen in the corresponding SEM image (compare Fig. 5a and e). The feedback measured on the bare zinc – 1 M ZnSO_4_ electrolyte interface is primarily positive (*i.e.*, *I*_T_/*I*_T,∞_ > 1), in contrast to the bare zinc map acquired in 2 M ZnSO_4_.

After the first plating (Fig. 5b), the surface becomes more conducting, evident by a transition from a deep purple colour to more blue and light blue regions on the surface. There is a very localised region of very localised high current (4.7 times the steady state current), seen in green and red. This may be indicative of a short due to the UME coming into contact with a surface feature. The corresponding SEM image (Fig. 5f) shows a region of shards that are more clearly defined than in the SEM image for the first plating in the 2 M ZnSO_4_ electrolyte (Fig. 4f). Whereas the 2 M ZnSO_4_ electrolyte seems to show small agglomerations of shards/flakes on the surface, the 1 M ZnSO_4_ shows more clearly defined hexagonal shards of zinc metal. Some of these shards are horizontally aligned, and some are vertically aligned, but the image seems to suggest a flatter surface, which could explain why the current distribution in the first plating of the 1 M ZnSO_4_ electrolyte is more homogeneous than that of the first plating in the 2 M ZnSO_4_ electrolyte.

The SECM image after the fifth plating shows a conductive surface (Fig. 5c), which is more homogeneous than the surface after the first plating. In the SEM image of the first plating (Fig. 5f), local growth of zinc metal is observed with some areas containing shards, but some areas are more dense and less flat with many small flakes. The corresponding SEM image of the fifth plating (Fig. 5g) shows that there are fewer of these dense and patchy regions, and a more homogeneous distribution of zinc shards on the surface. This contributes to a more homogeneous SECM image. The SEM image shows that as the electrode cycles, regions with more vertically aligned hexagonal zinc grows (Fig. 5g). These shards are approximately 1 μm in length, so not resolvable by SECM, since the UME has a spatial resolution of approximately 10 μm. Initial cycling in 1 M ZnSO_4_ leads to regions of vertically aligned zinc, with other regions that are elevated and denser (Fig. 5f), while cycling in 2 M ZnSO_4_ leads to the growth of agglomerated particles of metal (Fig. 4f).

The SECM image taken after the tenth plating (Fig. 5d) shows that the entire surface becomes more conductive, with regions of higher conductivity of two times the steady state current appearing on the map. The corresponding SEM image (Fig. 5h) shows large hexagonal shards of zinc that are approximately 10 μm in length, mainly vertically oriented, which should be resolvable by the UME. Similarly to the bare zinc SECM image (Fig. 5a), where the orientation of the SECM scan is different to that of the SEM scan, the shards observed in the SEM resulted in thin lines of higher current on the SECM image. This SECM image depicts a more conductive surface than the tenth plating in the 2 M ZnSO_4_ (Fig. 4d). Potentially, this leads to fewer hotspots and less risk of short circuit. The shards depicted in this SEM image are very thin and, therefore, less prone to pierce the separator.

## Electrochemical properties of a Zn||Zn symmetric cell

**Fig. 6:**
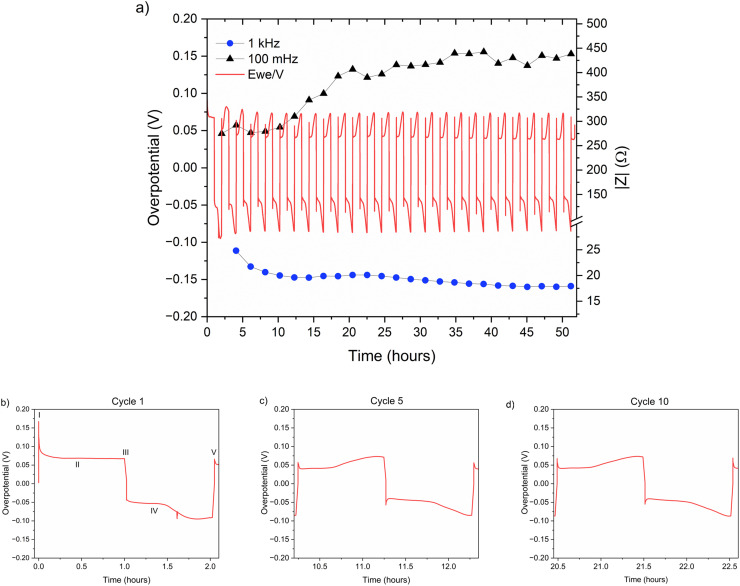
(a) Left *Y* axis: a voltage trace (*E vs. t*) of successive galvanostatic cycling of a total of 0.5 mA h cm^−2^ of zinc in a symmetric cell with 2 M ZnSO_4_ aqueous electrolyte and a Celgard 3501 polymer separator. Constant current density 0.5 mA cm^−2^ at 1C (plating and stripping 0.5 mA h cm^−2^, which is equivalent to 1.2 mg cm^−2^ zinc). Right *Y* axis: impedance modulus at 0.1 Hz (black), 1 kHz (blue). (b)–(d) Magnification of selected voltage traces (*E vs. t*) of successive galvanostatic cycling of a total of 0.5 mA h cm^−2^ of zinc in 2 M ZnSO_4_. Cycles 1 (b), 5 (c), and 10 (d) are presented. Characteristic peaks and processes are labelled (*I*–*V*), following the assignments made by Dasgupta *et al.*^34^

The zinc metal interface dynamics were next studied using symmetric zinc coin cells. To explore the effect of electrolyte concentration, galvanostatic plating and stripping cycles were conducted with currents of 0.5 mA cm^−2^ in 1 M ZnSO_4_ and 2 M ZnSO_4_ aqueous electrolytes, and 0.5 mA h cm^−2^ (4 mg cm^−2^) of zinc was stripped and plated per cycle (Fig. 6a). The galvanostatic periods were alternated with potentiostatic impedance measurements.

The shape of the voltage trace of the galvanostatic measurements is determined by the metal electrode morphology, the overpotentials developed in the cell, and the charge transport mechanism.^19^ Our analysis of the zinc voltage traces is based on the analysis of the related lithium plating and stripping voltage traces by Dasgupta *et al.*;^34,35^ a similar approach was used for the analysis of zinc plating voltage traces for the detection of soft shorts.^9^ During the first step (Fig. 6b), zinc is stripped from the zinc anode (the electrode where the electrooxidation process occurs), while metal nucleation occurs on the cathode (the electrode where electroreduction occurs). The first plating-stripping period starts with a peak in the overpotential, typically attributed to the overpotential required to overcome the activation energy barrier of metal nucleation (*η*_ac_) (Fig. 6b, labelled as I). Further zinc growth taking place on the cathode results in a voltage plateau (Fig. 6b, labelled as II). Due to a number of physical effects outlined above, this potential is not zero. After switching the current polarity, electrooxidation occurs from the zinc microstructures (composed of perpendicular shards and mossy zinc, see SEM images in Fig. 4 and 5). Interestingly, the voltage trace of the first striping has no nucleation peak (expected after the polarisation at III). At the same time, in the following cycles, a peak in the overpotential is observed. The voltage trace forms a plateau (IV) following the current direction switch (III). After stripping approximately half of the plated zinc, the overpotential increases, and the voltage trace forms an ‘arc’ shape, heading to a new plateau. Then, there is a drop in the overpotential due to the following current direction switch (V).

**Fig. 7:**
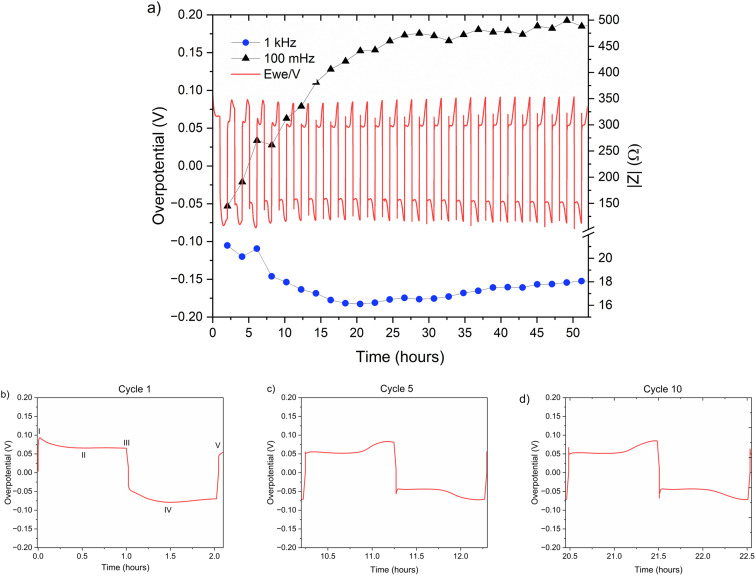
(a) Left *Y* axis: a voltage trace (*E vs. t*) of successive galvanostatic cycling of a total of 0.5 mA h cm^−2^ of zinc in a symmetric cell with 1 M ZnSO_4_ aqueous electrolyte and a Celgard 3501 polymer separator. Constant current density 0.5 mA cm^−2^ at 1C (plating and stripping 0.5 mA h cm^−2^, which is equivalent to 1.2 mg cm^−2^ zinc). Right *Y* axis: impedance modulus at 0.1 Hz (black), 1 kHz (blue). (b)–(d) Magnification of selected voltage traces (*E vs. t*) of successive galvanostatic cycling of a total of 0.5 mA h cm^−2^ of zinc in 1 M ZnSO_4_ aqueous electrolyte. Cycles 1 (b), 5 (c), and 10 (d) are presented. Characteristic peaks and processes are labelled, following the assignments made by Dasgupta *et al.*^34^

Dasgupta *et al.* showed that a similar voltage trace behaviour (*i.e.*, formation of a plateau that replaces the ‘double peaking’ voltage trace) in lithium cells is due to a new mass transport limitation which develops during prolonged cycling. The arc-shaped voltage trace is attributed to an additional overpotential, which develops due to the stripping of the freshly plated “dendritic” metal and the accumulation of “dead” metal (which creates a porous region), and potentially Zn-SEI. Interestingly, the voltage traces of the fifth and tenth cycles have nucleation peaks (Fig. 6c, d and 7c, d) and arc-shaped curves, which are assigned to the nucleation of fresh zinc on a passivated surface and to the development of a mass transport limitation, respectively.

To investigate zinc interface dynamics, the impedance modulus (denoted here as the impedance of the sample) measurements at 1 kHz and 0.1 Hz (which are widely assigned to the interface and charge transport impedance, respectively)^36^ and the voltage traces were plotted *vs.* the cycling time (Fig. 6a and 7a). The impedance modulus integrates the effective combined opposition to the passage of electrical current through a circuit due to its resistive and reactive components for each specific applied frequency. While the overpotentials of the 2 M and 1 M cells cycled at 0.5 mA cm^−2^ are not significantly different (compare Fig. 6a and 7a and see Fig. S28 and S30†), the impedance at 0.1 Hz grows approximately 6.5–18% per cycle in the 2 M cells and 14–16% per cycle in the 1 M cells during the first 25 cycles before both plateau (compare polymer separator-based cells depicted in Fig. 6a and 7a and GF-based cells in Fig. S24 and S26†). Though the comparison of three cells is not sufficient to determine the exact impedance growth rate, the broader distribution of the impedance growth per cycle in the 2 M cell is, potentially, indicative of the increased heterogeneity of the plating and stripping processes in 2 M ZnSO_4_.

The impedance at 1 kHz, on the other hand, exhibits a different trend: decrease and stabilisation for the 2 M cells (Fig. 6a and S28†), and decrease to a minimum and then a moderate increase for the 1 M cells (Fig. 7a and S30†). When the polymer separator is replaced with a GF separator, the impedance at 1 kHz is very low and constant for both concentrations (Fig. S24 and S26†).

While the single frequency impedance provides a convenient tool to track trends in the evolution of charge transport across the cell, the Nyquist plots provide further information on the charge transport mechanism (Fig. S33 and S34†). The plots may be interpreted as consisting of significantly depressed semi-circles, which suggests that even after the first plating, the surface of zinc becomes very rough and heterogeneous. Typically, this type of Nyquist plot is fitted using a constant phase element (CPE) rather than an ideal capacitor.^37^ Attempts to fit the EIS data with an equivalent circuit with components representing physical aspects of the cell that could be present were not successful, potentially due to the heterogeneous electrode morphology and SEI coverage. Equivalent circuits with many more components could possibly be used to fit the data, but no physical insight would be gained.

The electrochemical spectra in Fig. S33a† were measured after the first plating in 2 M and 1 M ZnSO_4_ with a polymer separator (the cycling is depicted in Fig. 6 and 7). The shape of the 2 M ZnSO_4_ spectrum after the first plating suggests a single interface and a time-dependent (not-steady state) semi-infinite linear diffusion, which is typical for the mass transfer of redox species to the electrode surface, while the 1 M spectrum shows a somewhat depressed semicircles. Importantly, the depressed semi-circle shape of the Nyquist plots after the first stripping is potentially a result of the decreased surface area rather than interphase formation. This is because when the surface area is high, the effect of ion diffusion in the electrode pores is more significant, and the contribution of mass transport in the bulk electrolyte to the total impedance is neglectable, as was shown for supercapacitor electrodes.^38^

After the 15th stripping (Fig. S33b†), the shape of all the spectra after stripping is more typical for a steady-state diffusion region of a finite length. However, at this stage, both electrodes are rough, and the total zinc surface area in the cell is not likely to change significantly between the cycling stages. This suggests that a new interphase was formed after the first cycle in the 1 M ZnSO_4_ and after 15 cycles in both electrolytes. To validate this finding and confirm that soft shorts were not formed during the cycling, the Nyquist plots after the fifth, tenth and fifteenth plating in 2 M and 1 M ZnSO_4_ electrolytes on the cathode were compared in Fig. S34.† The PEIS spectra in 1 M had a shape typical for a steady-state diffusion region of a finite length. In comparison, the spectra in 2 M had the shape of a single interface and a time-dependent (not-steady state) semi-infinite linear diffusion. Neither of these Nyquist plots is typical for a soft-shorted cell.

**Fig. 8:**
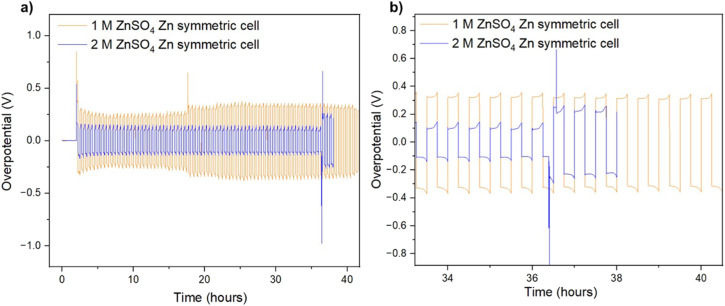
(a) Voltage trace (*E vs. t*) of successive galvanostatic cycling of a total of 2.75 mA h cm^−2^ of zinc at 11 mA cm^−2^ (4C) in zinc symmetric cell with 2 M ZnSO_4_ aqueous electrolyte, glass fibre separator. (b) Magnification of voltage trace (*E vs. t*) at the soft short formation.

It was recently demonstrated that a high cycling rate of zinc anodes depresses hydrogen evolution and promotes a higher efficiency compared to low currents.^39^ This high rate cycling was used here to compare the cyclability of the zinc anodes since high-rate cycling also has the potential to be strongly affected by SEI formation (rather than the competing hydrogen evolution process).^40^ Zinc symmetric cells with 1 M and 2 M ZnSO_4_ electrolytes and a glass fibre separator were cycled at 11 mA cm^−2^ (4C), cycling 2.75 mA h cm^−2^ zinc, for over 40 hours (Fig. 8a and S32†).^9^ The GF separator was used to prevent polarisation due to the overpotential contributed by the polymer hydrophobicity (Fig. S35†). The cells showed similar behaviour during the first 36 hours (Fig. 8), then the cell with the 2 M ZnSO_4_ electrolyte (blue voltage trace) underwent an erratic polarisation, followed by an overpotential increase, a behaviour typical for the formation of soft shorts (Fig. 8b).^19^ At the same time, the 1 M cell (orange voltage trace) overpotential initially decreased (due to the surface area growth) and then exhibited overpotential growth. This may be attributed to increased mass transport limitations, such as SEI accumulation. A similar trend was observed in duplicate cells (Fig. S32†), where the 2 M cell's voltage trace shape changed from a ‘peaking’ to a square shape was taken as an indication of soft shorts, as shown by Menkin *et al.*^19^

**Fig. 9:**
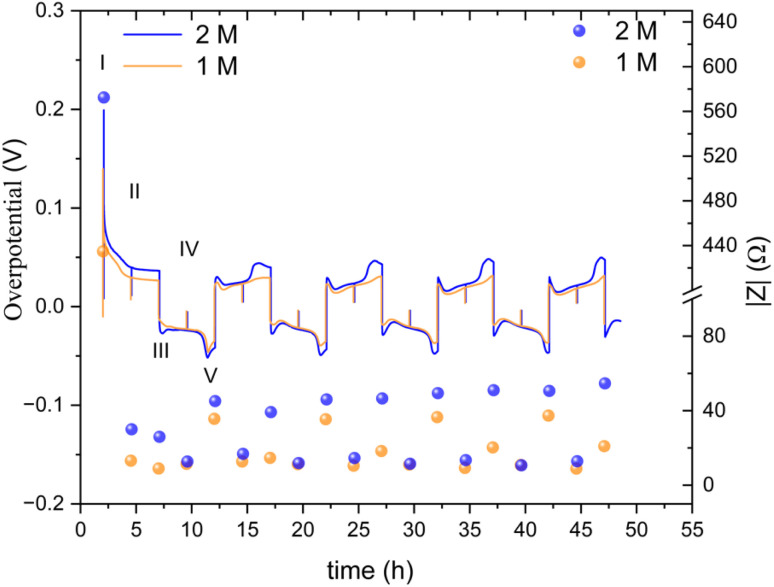
An overpotential trace of successive galvanostatic cycling of a total of 2.5 mA h cm^−2^ of zinc at 0.5 mA cm^−2^ (low rate, 0.2C) in zinc symmetric cell with 2 M and 1 M ZnSO_4_ aqueous electrolyte, GF separator, at 10 °C (left *Y* axis) and the total impedance at 10 Hz, measured during OCV (right *Y* axis). The galvanostatic impedance spectra (GEIS) were recorded every 2.5 hours, between 500 and 1 Hz with 100 μA amplitude. The single-spectrum measurement time was nine seconds. A drop in the overpotential (a spike in the overpotential trace) was observed when the GEIS measurements were taken at stages II and IV. Characteristic peaks and processes are labelled, following the assignments made by Dasgupta *et al.*^34^

The ionic transport rate is expected to decrease with temperature, whilst the electronic charge transport is not expected to be affected significantly if there were soft shorts, when comparing the charge transport rates at 25 °C and 10 °C. Hence, conducting soft short detection experiments at 10 °C is expected to highlight the effect of mass transport. To further understand the soft short formation mechanism, symmetric zinc cells were cycled at a lower current density (0.5 mA cm^−2^) at 10 °C. The lower operation temperature means that impedance measurements in which the main contribution is either ionic or electronic charge transport can be distinguished (Fig. 9).

The overpotential traces in 1 M and 2 M have comparable magnitude and shape. However, the overpotential in 1 M is consistently lower than in 2 M. This trend is especially apparent at the peaks typical at the beginning and end of the plating steps (III, V) when nucleation and bulk pitting introduce additional overpotentials to the system. Starting from the second cycle, the overpotential trace intensity, where the bulk pitting of the opposite electrode is expected (V), changes significantly into a shape previously assigned to hindered mass transport.^19,34,35^ This trend is more significant during plating on the cathode (the electrode on which was plated first).

A lower impedance and overpotential in (I) is attributable to lower initial interface resistance in 1 M over 2 M ZnSO_4_ (in contrast to the bulk conductivity trend, as will be discussed later). The impedance decreases linearly in the first step of the first cycle. At stage IV (the midpoint of the second of the first cycle) of the first cycle, the impedance of the 2 M cell reaches a minimum, while the 1 M impedance reaches its minimum at step III. From this point, both cells' impedance changes periodically with the cycling.

The impedance remains minimal at steps II and IV, thereafter. At the V steps, the impedance increases and reaches a local maximum. The local maximum values slowly increase with the cycle number for the 2 M. The impedance measured at stage III (after the first step of each cycle) is significantly lower in 1 M. The 1 M cells showed a much slower increase in the impedance measured at stage V. We attribute this gentle impedance increase between the cycles to additional mass transport overpotential development. Hence, in contrast to the 1 M cell, stripping the freshly plated zinc from the cathode in the 2 M cell is insufficient to decrease the impedance to its minimal value. Comparing the voltage traces, the nucleation overpotential and the overpotential ‘arc’ that is typically attributed to mass transport limitations, are higher in the 2 M electrolyte. Alternatively, this ‘arc’ is formed due to the accumulation of ‘dead’ zinc.

The periodic nature of the impedance evolution throughout the cycling is apparent in the GEIS spectra (Fig. S36 and S37†). While both 1 M and 2 M cells showed a periodic trend in both sets of data (Fig. 9, S36 and S37†), the growth of the impedance of the 2 M cell is faster. The Nyquist plots at stages III and IV, when one of the electrodes is plated with an amount of 2.5 mA h cm^−2^ and the other stripped off the same amount of zinc, have more capacitive nature, evident in the higher values of *Z*′′, than the spectra at stages II and IV. None of the Nyquist plots are typical for a soft short, however the significant impedance decreases in stages II and IV is attributed to potentially periodically reoccurring reversible soft shorts.

High-rate cycling (11 mA cm^−2^, 4C) was done at 10 °C to highlight the effect of mass transport (Fig. S38 and S39†). Increasing the current density (or lowering the temperature) is expected to decrease the concentration of zinc ions near the surface and lead to dendritic deposition.^39,40^ However, the high-rate cycling at low temperature was more stable compared to the room-temperature cycling (Fig. 8), and in contrast to the low rate, the cycling and the impedance measurements in 1 M and 2 M ZnSO_4_ are relatively similar. Interestingly, here, the voltage traces of the first cycles, in 1 M and 2 M ZnSO_4_, have arc shapes which evolve into a double peaking shape, the opposite trend compared to alkali metals. The 10 Hz impedance trends indicate potential reoccurring soft shorts and interface impedance stabilization for both concentrations. However, consistently with the low-rate experiment (Fig. 9), the impedance increase is less reversible between the different cycle stages for 2 M ZnSO_4_.

## Raman

To help understand the Zn-SEI formation mechanism, the effect of ZnSO_4_ concentration on Zn-SEI composition was investigated. Raman spectra of the reference samples, ZnO and ZnSO_4_·7H_2_O, are shown in Fig. S41 and S42.† The main bands of ZnSO_4_·7H_2_O due to the symmetric stretching (ν_1_), bending (ν_2_, ν_4_) and deformation (ν_3_) of SO_4_^2−^ are denoted in Fig. S42.† A broad band is observed at 388 cm^−1^ due to symmetric stretching of (Zn–O) of octahedrally coordinated hexahydrate [Zn(H_2_O)_6_]^2+^. Similarly, the main bands of ZnO are assigned in Fig. S41.† The spectra of ZnSO_4_ solutions also show the band corresponding to [Zn(H_2_O)_6_]^2+^, which is more prominent in 2 M ZnSO_4_ than 1 M ZnSO_4_ with a slight red shift of the ν_1_-SO_4_^2−^ band (Fig. S43 and S44†).

**Fig. 10:**
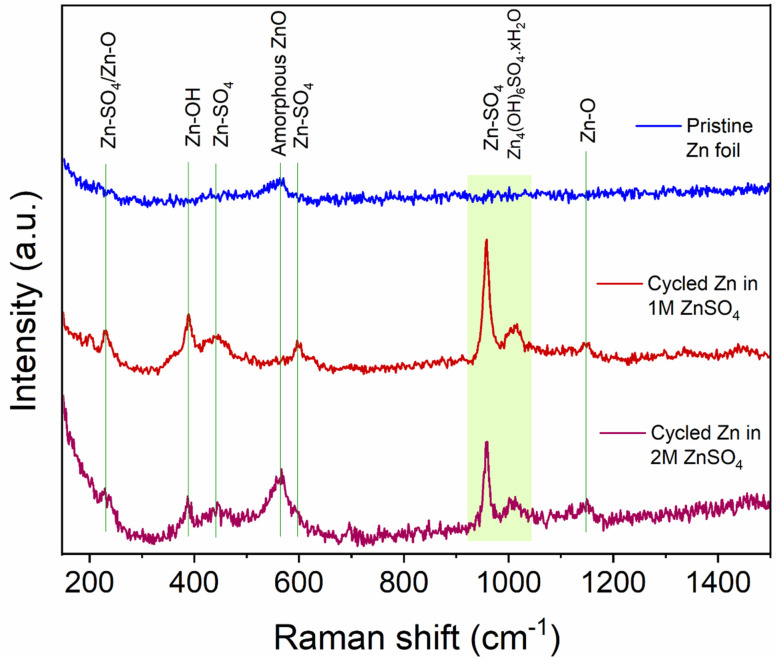
Comparison of Raman spectra of bare and cycled Zn electrodes. The assignments of key bands are made based on reference samples (Fig. S41–S45†). The electrodes were cycled for 40 h (20 cycles) in 1 M and 2 M ZnSO_4_ electrolytes with a Celgard 3501 polymer separator. Samples were washed with distilled water prior to measurement.

The Raman spectra of the zinc electrodes after 40 hours of plating and stripping in a Zn||Zn symmetric cell in 1 M and 2 M ZnSO_4_ electrolytes are compared with bare zinc foil in Fig. 10. The bare foil does not show signatures of any species except for a very broad band at 565 cm^−1^ corresponding to amorphous ZnO. However, this band is not observed at all the positions of the bare foil, indicating heterogeneity of the ZnO layer. The cycled zinc electrodes show various bands corresponding to Zn–SO_4_ and Zn–O species, as assigned in Fig. 10, considering reference samples as discussed above. The positions of these bands suggest the presence of a species with reduced SO_4_^2−^ symmetry on the surface, giving rise to the highest intense band around 958 cm^−1^. Thus, the assigned Zn–SO_4_ and Zn–O bands are a result of this reduced symmetry species. These findings confirm the presence of Zn_4_(OH)_6_SO_4_·*x*H_2_O on the surface of cycled Zn electrodes in 1 M and 2 M ZnSO_4_ solutions. The intensities of all the bands corresponding to this species are higher for the zinc cycled in 1 M ZnSO_4_ solution compared to 2 M analogue. Hence, qualitatively, the concentration of this species is higher on zinc cycled in the 1 M ZnSO_4_ solution compared to the 2 M analogue.

## XPS

X-ray photoelectron spectroscopy (XPS) measurements were performed to investigate the elemental composition and local environment of the bare zinc foil, as well as the compounds formed on the zinc anode. Here, the HR-XPS Zn-2p and S-2p regions are focused on resolving the zinc and sulphur chemical states and bonded groups. The atomic concentrations measured on the electrodes show an increasing content of sulphur-containing species, with an increasing concentration of ZnSO_4_ in the electrolyte. The slightly higher binding energy of bare zinc foil that was observed (compared to a bare zinc metal) can be attributed to zinc foil forming a thin layer of ZnO/Zn(OH)_2_ on its surface, as expected. The high carbon content in all the electrodes is attributed to the carbon present on the bare zinc foil. The adsorption of carbon and oxygen containing compounds to the surface can also take place because of XPS beam damage.^41^ The sulphur and zinc atomic percentage increases with a higher concentration of ZnSO_4_ in the electrolyte likely due to the higher amounts of zinc salt present at the interface (Fig. 10).^42–44^

**Fig. 11:**
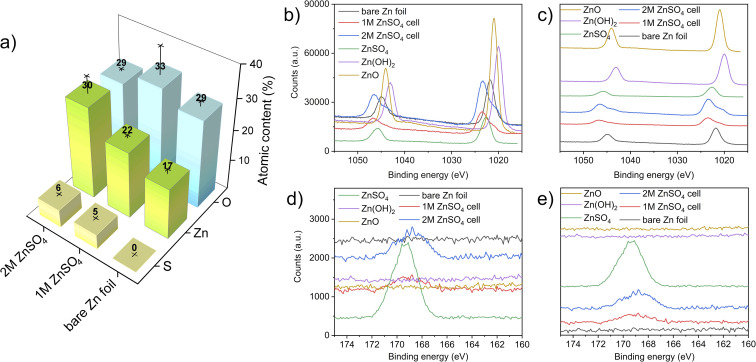
(a) Atomic concentration bar graph, where the atomic composition presented was derived from two measurements, taken at two different points of each analysed sample (bare zinc, cycled electrode in 1 M and 2 M ZnSO_4_), and the standard deviations are represented by lines and crosses. Panels (b) and (c) show the HR-XPS of the Zn 2p regions in overlay and offset presentation, respectively. Panels (d) and (e) show the HR-XPS of the S 2p regions in overlay and offset presentation, respectively.

The HR-XPS of the Zn 2p region revealed that the zinc environment of the two electrodes has a very similar binding energy to ZnSO_4_. Interestingly, the shoulders at lower binding energies at both the 2p_1/2_ and 2p_3/2_ of the electrodes from 1 M and 2 M cells can also be assigned to a ZnO-like environment, further confirming the heterogeneous nature of the interface formed on the electrodes. These results are congruent with the Raman analysis (Fig. 10), confirming both the sulphate-like and ZnO-like environments on the plated interface. Importantly, the relative intensity of both these peaks agrees with the atomic concentration results (Fig. 11a).

The HR-XPS of the S 2p region shows a broad signal centred around 168 eV for both the 1 M and 2 M ZnSO_4_ cells, indicating the presence of sulphate groups associated with ZnSO_4_, as the higher section of the broad signal agrees with the S 2p signal of a reference ZnSO_4_ salt. In addition, a small shoulder at the lower binding energies may imply a content of Zn_4_(OH)_6_SO_4_·*x*H_2_O, which was also found using Raman spectroscopy.^45–49^

Comparing the zinc–oxygen and zinc–sulphur ratios (Table S1†), it is shown that the layer formed on the electrode surface is more zinc-rich than the SEI composition in the literature, potentially due to the heterogeneous high surface area morphology of the plated zinc (Fig. 4 and 5) which facilitates the measurement of the exposed zinc under the SEI-free areas.^48,49^

## Electrolyte conductivity

Conductivity measurements were undertaken to investigate the effect of bulk electrolyte conductivity on the observed phenomena and to determine whether the conductivity change with the concentration introduces a potential limitation to the interface methods in this study. Conductivities of 0.2 (10.8 mS cm^−1^), 1 (29.5 mS cm^−1^), and 2 M ZnSO_4_ solution were measured, and 2 M was shown to have the maximal conductivity value of 46.7 mS cm^−1^. This compares to the conductivity trends measured by Hinatsu *et al.*^50^

## Discussion

The formation and the role of the SEI on zinc metal electrodes is one of the remaining questions in the study of ZMBs. There are notable similarities between zinc and alkali metal SEI formation and plating mechanisms. Similarly to alkali metals, zinc plating takes place outside the stability range of the aqueous electrolyte; however, while the reduction products of the organic electrolyte are typically solid, the aqueous electrolyte electrolysis results, in addition to the solid products, in parasitic hydrogen evolution and a local pH increase. This pH increase drives the formation of hydroxide and oxide-containing compounds (demonstrated using Raman and XPS, Fig. 10 and 11), which accumulate on the surface, forming an SEI-like layer. The fundamental difference is that since Zn-SEI forms simultaneously to zinc plating, it is not essential to the start of electrodeposition. However, since it was demonstrated in this work that zinc plating is surface controlled, similarly to alkali metals (as will be discussed later), zinc can be treated as an SEI-forming metal.

SECM scans of the pristine zinc foil unveil a slight heterogeneity in the low current range, while SEM demonstrates slight changes of topology on the surface (Fig. 4a, e and 5a, e). This is potentially due to the carbon-containing compounds which were found on the bare zinc and on the cycled samples in the XPS study (Fig. 11 and S40†). The carbon on the bare zinc is possibly a residue of the ethanol and acetone mix used for the cleaning of the zinc electrode.

The effect of surface heterogeneity of the metallic substrate on the morphology of electrodeposited metal in aqueous electrolytes has been widely studied, typically in the context of practical nickel electrodeposition.^51^ Here, it was demonstrated that heterogeneity of the zinc surface results in a heterogeneous electrodeposition and depending on the conditions (such as electrolyte concentration and/or current density) this then leads to a variation of roughness morphology. However, the heterogeneity of the bare zinc is likely to result in uneven nucleation regardless of the electrolyte composition and plating conditions, as was demonstrated by Zhao *et al.*^31^

In this study, different concentrations of ZnSO_4_ aqueous electrolytes, which were reported by others to be different by electrolyte solvation structure, were compared, determining the extent of hydrogen evolution and zinc cycling kinetics and efficiency.^24^

SECM was used to explore the electrochemical heterogeneity of the interface, which was formed and evolved in 2 M ZnSO_4_. PAC measurements indicate that even after one plating, the surface of a zinc metal anode becomes electrically insulating. This suggests that an electrically insulating interphase is formed as a heterogeneous SEI-like layer, possibly because of the lower hydrogen evolution. Alternatively, it could be due to plating more horizontally aligned zinc or local passivation, which would lead to a negative feedback case being observed, if it is assumed that horizontal zinc forms ZnO more easily.^31^

The PAC after the tenth cycle (Fig. 3, green line) shows behaviour that deviated from the pure negative feedback case, as the tip current decreases faster than the other recorded traces, and then increases and finally decreases again as it approaches the surface. This behaviour could result from the current measured *via* the UME, which travels vertically through the deposited materials, besides zinc shards, and senses regions of electron-conducting and non-conducting electrode surfaces. The somewhat ambiguous conclusions demonstrate that a single PAC measurement is insufficient for characterising a potentially heterogeneous surface. This was confirmed by SECM and SEM images (Fig. 4a–h). After ten cycles, these protrusions seem to agglomerate into bigger features, that increase the roughness of the surface (Fig. 4h). The roughness and heterogeneity are also typical to cycle five, but this trend seems to change at cycle ten, when the surface becomes more electrochemically homogeneous. SEM showed that for 2 M ZnSO_4_, the morphology is rough and dominated by protruding agglomerations of zinc metal (Fig. 4h), which could potentially lead to more exposed zinc surface (higher atomic concentration) and less ZHS accumulation (as seen in Raman and XPS measurements). The combination of the morphology and the heterogeneous ZHS coverage leads to a higher tendency to form hotspots and therefore a higher tendency to short, leading to a shorter cycle life.

PAC measurements over an electrode cycled in 1 M ZnSO_4_ unveiled a different story to that of the electrode cycled in 2 M ZnSO_4_. The zinc electrode exhibited positive feedback behaviour after the first plating cycle (Fig. 3, turquoise line). This indicated that in the region that was approached, an electrically insulating interphase had not yet accumulated to an extent enough to block the transfer of electrons. However, the probe approach curves recorded after the fifth and tenth plating demonstrate negative feedback (Fig. 3, black and purple lines), indicative of the accumulation of an electrically insulating interphase. Cycling the cell would allow the build-up of both passivating elements such as ZnO and Zn(OH)_2_, whereas hydrogen evolved during cycling causes local pH changes and the production of ZHS. Therefore, it is suggested that the Zn-SEI accumulates between cycles one and five for 1 M ZnSO_4_ electrolyte system. The SECM images of the tenth plating in 1 M ZnSO_4_ (Fig. 5d) depict a more conductive surface than the tenth plating in the 2 M ZnSO_4_ (Fig. 4d). Potentially, this leads to fewer hotspots and less risk of short circuit. The shards depicted in the SEM images (Fig. 5b–d) are very thin and flexible and, therefore, less prone to pierce the separator.

In 1 M ZnSO_4_ there is reported to be more hydrogen evolution, and hence more OH^−^ formation, which potentially leads to more ZHS formation as demonstrated with Raman (Fig. 10). This would encourage better, more complete ZHS coverage, leading to a lower zinc atomic concentration, which was demonstrated with XPS (Fig. 11). The morphology in these samples is dominated by zinc shards which grow with the cycle number and are typical for zinc plating when the hydrogen evolution is significant.^52^

While the morphology results in primary current distribution (*i.e.* the effective bulk electrolyte resistance is lower), the interphase heterogeneity determines the secondary current distribution (*i.e.*, the local resistance of the interface determines the local current).^53^ The dynamic overpotential recorded during cycling at low rates (Fig. 6–9) serves as an additional demonstration of the combined effect of the heterogeneity of zinc morphology and the uneven ZHS interphase. The stripping takes place preferentially from areas on the electrode where the overpotential is minimal. Herein, the continuous stripping of the porous zinc electrode which contains “hidden” interfaces and various levels of roughness results in an arc-shaped overpotential which is shown in Fig. 6b–d and 7b–d. This effect is more pronounced during cycling in the 2 M ZnSO_4_ electrolyte at 10 °C.

Our findings demonstrated that surface processes have a significant impact on zinc plating. Additional cycling experiments were performed to establish whether bulk or surface processes affect zinc cyclability more. In the former case, using 2 M ZnSO_4_ should result in more stable cycling than 1 M ZnSO_4_. While at low-medium rates (1C), the cyclability of 2 M ZnSO_4_ and 1 M ZnSO_4_ is similar, when cycling at high rates (4C), there are significant advantages to cycling with 1 M ZnSO_4_ since the 2 M electrolyte showed significant polarisation and soft shorts after a shorter cycling time. The longer cycle life at 4C of the 1 M cells is attributed to the more conductive SEI and the presence of fewer hotspots, as unveiled by SECM. However, high-rate cycling of the zinc anode in 1 M and 2 M ZnSO_4_ at low temperature (10 °C) gave rise to ambiguous results, potentially due to a more dominant concentration polarisation at the low temperature that negatively affected the cyclability in 1 M ZnSO_4_ (in lower concentration the ion depletion near the electrode is more critical). The *in situ* GEIS showed that while the tendency for reversible soft shorts is similar, impedance increase between the cycling stages is more significant and less reversible in 2 M ZnSO_4_, consistently with the overall trend of surface-controlled degradation on the zinc anode.

A closer examination of the voltage traces for the first cycle (Fig. 6b and 7b) show that the typical overpotential maximum at the beginning of the plating, which is assigned to the metal nucleation, is missing from the first stripping. This shape of the voltage trace is typical for plating in SEI-dominated systems, where the electrolyte reduction or electrode alloying^51^ overpotential is significantly higher than the nucleation overpotential and hence masks it. However, since ZHS does not form a full coverage of the anode, this mass transport-dominated voltage trace, seen in all the experiments, is more likely to result from the porous morphology that develops during the first cycles. The additional mass transport overpotential (due to the increased tortuosity of the zinc electrode) adds to the total overpotential and results in the masking of the relatively small nucleation peak.

The impedance at 0.1 Hz is the accumulative impedance of all cell components (*i.e.* total impedance). Hence, the response at 0.1 Hz was assigned to the evolving mass transport barriers because of the formation of an SEI-like interphase. Although there is some evidence for interphase formation on the first cycle (due to the voltage traces in Fig. 6b and 7b, typical for plating on a formed SEI) the low-frequency impedance grows significantly from the first to the 15th cycle. A change in the shape of Nyquist plots (Fig. S33 and S34†) suggests a transition from a semi-infinite ion diffusion into the freshly deposited porous zinc to a diffusion in the finite length solid layer on the surface. However, further studies of the ZHS compounds and formation mechanism are required.

In alkali metal systems, the impedance at 1 kHz is commonly attributed to the charge transport across the interface (*i.e.* the SEI) and evolves with the morphology and SEI growth; thus, if Zn-SEI behaved similarly, its 1 kHz impedance should increase if SEI growth were more dominant and decrease if the surface area growth were more dominant, regardless of the separator used, as Menkin *et al.* previously demonstrated.^19^ However, the trend of the 1 kHz impedance does not agree with these trends. The initial decrease, followed by a stabilisation of the 1 kHz-impedance for the polymer separator cells (Fig. 6a and 7a) *vs.* the constant low impedance of the GF cells (Fig. S24–S27†), is potentially due to the hydrophobicity of the polymer separator and the hydrophilicity of the glass fibre separator. It is suggested that the measured EIS at 1 kHz is dominated by ion transport in the zinc electrode pores and the wetting of the polymer separator rather than zinc SEI growth. To validate these assumptions, separator wetting experiments were conducted, showing significantly better wetting of the GF separator by the aqueous electrolyte (Fig. S35†).

The critical role of the separator in zinc cells was previously discussed in the literature.^54,55^ The most common separator used in research, a glass fibre (GF) separator, has irregular pore sizes and fragile structures incapable of resisting the zinc metal dendrites, which may grow along the pores or penetrate the GF separator, and cause rapid short circuits, dead zinc and rapid degradation.^54^ Celgard 3501 separator (a polyethene–polypropylene film with a silica coating) is used for zinc cells as well.^56^ However, it is relatively hydrophobic and, therefore, suffers from wetting issues. Moreover, since Celgard is also a porous separator, it has been shown to facilitate short circuits like the GF separator.^55^

In this study, the role of the separator was highlighted, comparing separator-free zinc plating in an *in situ* SECM cell designed during this study and coin cells with either polymer or a GF separator. While the coin cells provide a more realistic setup, using a separator-free cell is more suitable for fundamental studies. Indeed, the intricate zinc structures revealed during the SEM study will likely be depressed by contact with the separator or during coin cell assembly. When comparing the coin cell results, the glass fibre cells have a lower overpotential (Fig. S24–S27†), however, show a less consistent trends and tend to form soft shorts. Hence, while the polymer separator-based cells have significantly higher overpotentials and potentially suffer from poor wetting of the hydrophobic separator, using the porous GF separator introduces a more significant short-circuit risk.

In this paper, symmetric cells were used to focus and highlight the role of the anode in ZMBs, however, cycling of full cells for both electrolyte concentrations are also demonstrated in Fig. S54.† The electrode balancing and cycling profile should be further optimised to achieve prolonged cycling.

While having the electrochemical model in hand, the composition of the Zn-SEI using Raman and XPS spectroscopies was investigated. Raman and XPS measurements showed that the SEI-like layer formed on zinc electrodes after ten cycles is ZHS-based. However, the XPS analysis showed a higher concentration of zinc than expected from ZHS. This result is attributed to the heterogeneous and porous zinc plating that was demonstrated in 2 M and 1 M ZnSO_4_ electrolytes (Fig. 4 and 5), which allows the measurement of the underlying zinc metal.

The heterogeneity of the bare zinc surface was demonstrated *via* a higher binding energy of the bare Zn. The significant presence of carbon-containing compounds on the surface suggests a potential plating mechanism that encourages the growth of heterogeneous zinc protrusions and shards during the first cycles (although it is noted that carbon is usually present in XPS measurements as a contamination). These hypotheses agree with the SECM analysis, which showed that the zinc interface is significantly more heterogeneous after the first and the fifth cycles, compared to the tenth cycle, after which the interface became more homogeneous (Fig. 4 and 5). The increase of the electrochemical homogeneity of the interface was more significant in the 1 M ZnSO_4_ electrolyte (Fig. 5). The latter result agrees with the higher Raman signals of ZHS (Fig. 10) and the superior cycling in the 1 M ZnSO_4_ electrolyte (Fig. 8).

Here, the formation of an SEI-like layer on zinc *via* the reaction of ZnSO_4_ with hydroxide, formed on the surface due to local pH increase during hydrogen evolution is validated. On the one hand, this Zn-SEI is expected to slow down zinc anode degradation since the coverage of the metal surface is expected to inhibit hydrogen evolution and potentially the vertical zinc growth. On the other hand, the Zn-SEI formation process is expected to be more intense on the freshly exposed plated zinc and is formed because of hydrogen evolution and consequently encourage degradation. While prolonged cycling in either of the electrolyte concentrations can potentially result in excessive SEI accumulation and anode degradation, the increased heterogeneity of the zinc interface in 2 M leads to more rapid degradation *via* soft shorts formation.

Importantly, these findings are not in contradiction to the recent studies showed a significant advantage of concentrated electrolytes in which there is less water in the Zn-ion solvation shell and as a result the cycling efficiency, which is dominated by hydrogen evolution, is significantly higher.^24^ Moreover, as many of the recent studies^57^ are focused on zinc plating on various metal current collectors (such as copper and titanium), the cycling of these cells is determined by the extent of hydrogen evolution and potential galvanic corrosion, which is dictated by the substrate surface chemistry. However, this study used symmetric zinc cells and aimed to explore the future of zinc metal batteries.

This work demonstrated that while the ZHS-based SEI results in partially horizontally oriented zinc plates in 2 M ZnSO_4_, as was shown by Zhao *et al.*,^31^ the zinc metal orientation in 2 M is sometimes horizontal, while in 1 M it is primarily vertical. Hence, ZHS accumulation does not guarantee horizontal orientation of zinc (Fig. 4, 5 and 10, 11). However, the topography of the electrode, determined by the structure of the zinc agglomerates, is significantly rougher in 2 M, resulting in a higher tendency for soft shots and shorter cycling.

Although zinc electrodeposition is widely studied, whether the surface or the bulk charge and mass transport processes control the electrodeposition has yet to be determined. Here, we showed that the topography of the zinc affects zinc cyclability more significantly than SEI composition and electrolyte conductivity. Although 2 M ZnSO_4_ has higher conductivity than 1 M and the composition of the SEI is very similar between the two electrolytes, zinc anode tendency for soft shorts is more significant in 2 M due to the typical rough and porous morphology and increased interface heterogeneity of the plated zinc.

## Conclusions

ZMBs present a more sustainable alternative to today's lithium-ion batteries for stationary applications. The most significant advantage of ZMBs is the aqueous non-fluorinated electrolyte, which is significantly less toxic, less flammable, and lower cost compared to lithium or even sodium ion electrolytes. However, this aqueous electrolyte introduces the biggest challenge in developing ZMBs: controlling parasitic electrolyte reactions on dynamic, high surface area, and reactive metal electrodes.

Exploring the dynamics of zinc interface electrochemistry with SECM, EIS, Raman and XPS, this work showed that an SEI-like layer composed of mainly ZHS forms on the surface of zinc during Zn-ion reduction, arising from hydrogen evolution and local pH increase. The hydroxide ions react with zinc metal and ZnSO_4_, forming ZHS. The amount and heterogeneity of the precipitated ZHS are affected by Zn-ion concentration.

The chemical and electrochemical heterogeneity of the zinc interface results in a heterogeneous nucleation of zinc. However, the morphology of zinc and its degradation during cycling is determined by the local electrochemical processes at and near the interface, which are influenced by the competing Zn^2+^ and water reduction processes. This research has shown that manipulating the electrolyte concentration can influence this competition. Lower electrolyte concentration encourages more evenly distributed hydrogen evolution and zinc SEI formation, leading to more even topography and more homogenous electrochemical reactivity, which result in more extended high rate cycling and a reduced tendency for soft shorts. However, further research should be done to increase the efficiency of the 1 M ZnSO_4_-based ZMBs.

The key difference between alkali metals and zinc plating is that SEI healing governs the morphology and efficiency of the cycling of alkali metals. In contrast, in zinc systems, local electrolyte reactivity at the metal-electrolyte interface determines the cell's efficiency and longevity.

This SECM study, conducted under realistic battery cycling conditions, has shed light on the crucial role of interface homogeneity in zinc metal batteries and the necessity of scanning probe electrochemical methods for the comprehensive study of metal batteries. This work showed that SEI charge transport properties have more significant effect on zinc anode cyclability compared to the tendency for hydrogen evolution or even electrode morphology.

Beyond zinc batteries, the developed methodology is transferable to the degradation studies of lithium-ion batteries and potentially can be used to enable lithium metal batteries and more sustainable chemistries such as sodium and calcium batteries.

## Experimental section

### Chemicals

ZnSO_4_·7H_2_O (99%) and ferrocenemethanol (≥97%) were purchased from Sigma-Aldrich and were used as received. Zinc foil (≥99.95%, 250 μm thickness) was purchased from Goodfellow Advanced Materials and was sonicated in a mixture of ethanol/acetone before use. A 10 μm Pt ultramicroelectrode (UME) was purchased from CH Instruments.

### Galvanostatic cycling

Zn||Zn symmetric cells with a glass microfibre separator soaked in 100 μL of 1 M and 2 M ZnSO_4_ were cycled with a capacity of 0.5 mA h cm^−2^ on a Biologic MPG2 potentiostat at either 0.5 mA cm^−2^ (1C) or 11 mA cm^−2^ (4C).

### Electrochemical impedance spectroscopy

PEIS was measured at a frequency range of 7 MHz to 100 mHz with an amplitude of 5 mV using a Biologic VSP-300 potentiostat. PEIS was measured using a Zn||Zn symmetric cell using a Celgard Monolayer Microporous Membrane (Celgard 3501), soaked in 100 μL of electrolyte. The symmetric cells were cycled at a capacity of 0.5 mA h cm^−2^ and PEIS was measured after every plating step.

### Electrolyte conductivity measurements

Solution conductivity measurements were made in a TSC 1600 Closed cell from RHD Instruments. 1 mL of each solution was filled into the cell. Impedance spectra were measured at room temperature (20 °C) using a PalmSens4 potentiostat, with an applied voltage amplitude of 20 mV (2 M and 1 M ZnSO_4_) or 50 mV (0.2 M) and frequencies between 1 MHz and 1 Hz. The impedance spectra were fitted using the equivalence circuit R + Q, and the solution conductivity was found by taking the reciprocal of the R component, multiplied by the cell constant.

The cell constant was determined using a 1413 μS cm^−1^ conductivity standard solution from Hanna Instruments. 1 mL of the solution was filled into the cell, which was sealed and placed in an incubator held at 25 °C. An impedance spectrum was measured as above. The spectrum was fitted with a Q + R/Q circuit and the measured resistance was multiplied by 1413 μS cm^−1^ to determine the cell constant, which was found to be 6.1 ± 0.1 cm^−1^

### Scanning electrochemical microscopy

Probe approach curves and scanning electrochemical microscope images were obtained using a CHI920C potentiostat from CH Instruments. Each of these experiments was performed using a 4-electrode cell consisting of a 10 μm Pt UME working electrode (imaging and probe approach), a 16 mm zinc foil working electrode (plating and stripping), a Pt wire counter electrode and a saturated Ag/AgCl reference electrode (3 M KCl). The zinc foil (Goodfellow) working electrode was electrically connected to a copper wire using a copper mesh current collector so the cell could be cycled. The foil used in the *in situ* SECM cell is comparable to the size used in a coin cell and allows a realistic mass loading. Approach curves and SECM images were obtained using a redox mediator solution consisting of 1 mM FcMeOH as a mediator and 0.1 M ZnSO_4_ as a supporting salt.

### FB-SECM images

SECM images were obtained for both a pristine zinc foil substrate and subsequent electroplated zinc on cycles 1, 5, and 10 with a capacity of 1.7 mA h cm^−2^. The UME was moved along the substrate at a constant height of 25 μm. The images were taken using an increment distance of 10 μm and an increment time of 5 s, yielding a scan rate of 2 μm s^−1^. The tip current was normalised by the steady state current and reflects the electrochemical heterogeneity of the substrate upon cycling. Images were collected using a UME potential of +0.5 V *vs.* Ag/AgCl.

### Probe approach curves

Probe approach curve (PAC) measurements were used to determine the standard heterogeneous rate constant of electron transfer at the surface. PAC measurements were obtained using a UME potential of +0.5 V *vs.* Ag/AgCl and measuring the resultant current as a function of distance from the substrate. Results are reported using a normalised tip current, where the measured UME current is normalised by the steady state current. The UME was approached towards the surface at a scan rate of 1 μm s^−1^.

### XPS

XPS analysis was performed on an EscaLab 250Xi (Thermo Fisher Scientific) with an Al Kα source (photon energy of 1486.7 eV; spot size of 400 μm). The obtained data was evaluated by using Avantage software (version 5.9 and 6.0). All spectra were charge-shifted, using the adventitious carbon peak (284.8 eV) as a reference. Data used in the HR-XPS graphs were used without scaling to preserve all the information and not to process them extensively. A piece of approximately 3 × 5 mm was cut from each sample and washed with distilled water for 10 seconds before being mounted on the sample holder. The atomic composition values were rounded to the nearest whole number.

### Raman

Raman measurements were performed using a confocal Raman microscope, Horiba LabRam HR Evolution equipped with a solid state 532 nm laser and diffraction grating 600 g mm^−1^. A 50× long working distance objective was used to focus the samples. The measurements were performed between 150 and 3800 cm^−1^ with 5 accumulations and 30/40 s acquisition time. The reference solid samples and the electrolytes were spread on a glass slide and covered with a microscope cover slip for the measurements. A small piece of electrode cut from the pristine and cycled Zn electrodes after cleaning were used for Raman measurements, which had been washed with distilled water.

### Electrode preparation and full cell studies

The slurry for the cathode was prepared by mixing 70 wt% of Mn_2_O_3_ (325 mesh, Aldrich), 20 wt% of acetylene black and 10 wt% of polyvinylidene fluoride (PVDF) in a 7 : 2 : 1 in *N*-methyl pyrrolidone (NMP) and coated on a stainless-steel current foil with the help of a doctor blade. The coating was dried at 60 °C for 12 h initially and then at 120 °C for 20 h under vacuum. Finally, 12.7 mm cathode discs were punched out of the foil to utilise those in the full cell studies with zinc metal. The active material loading is ∼2 mg cm^−2^. Coin cells were assembled with a piece of glass fibre separator and 100 μL of either 1 M or 2 M ZnSO_4_ as the electrolyte. All the measurements were conducted in the voltage range of 0.8–1.8 V *vs.* Zn metal.

## Data availability

The data supporting this article have been included as part of the ESI.† Details on the ultra-microelectrode choice and characterisation; additional probe approach curves formed at different experimental conditions, zinc plating and stripping curves recorded during SECM experiments, additional EIS data, a photo of separator wetting with the studied electrolytes, additional XPS data and Raman spectra, additional SEM images and full zinc battery cell cycling data. Data for this article are available at Apollo – University of Cambridge Repository at DOI: https://doi.org/10.17863/CAM.111425.

## Author contributions

JTS: conceptualisation, data curation, formal analysis, investigation, methodology, project administration, validation, visualisation, writing (original draft preparation), writing (review and editing). VS: investigation, data curation, visualization, writing – original draft, review and editing. DT: investigation, data curation, visualization, writing – original draft, writing – review and editing. HES: investigation and formal analysis. CPG and SMC: supervision, review and editing. SM: conceptualisation, formal analysis, investigation, writing (original draft, review and editing), supervision.

## Conflicts of interest

There are no conflicts to declare.

## Supplementary Material

TA-012-D4TA03165B-s001
