# Correction: The effect of interface heterogeneity on zinc metal anode cyclability

**DOI:** 10.1039/d4ta90166e

**Published:** 2024-09-09

**Authors:** J. T. Simon, V. Šedajová, D. Tripathy, H. E. Smith, S. M. Clarke, C. P. Grey, S. Menkin

**Affiliations:** a Yusuf Hamied Department of Chemistry, University of Cambridge Lensfield Road Cambridge UK sm2383@cam.ac.uk; b Institute for Energy and Environmental Flows, University of Cambridge Madingley Road Cambridge UK; c The Faraday Institution, Quad One, Harwell Science and Innovation Campus Didcot UK

## Abstract

Correction for ‘The effect of interface heterogeneity on zinc metal anode cyclability’ by J. T. Simon *et al.*, *J. Mater. Chem. A*, 2024, DOI: https://doi.org/10.1039/d4ta03165b.

The authors regret that the original article contained incorrect titles in the top line of Fig. 5; these read ‘2 M ZnSO_4_…’ instead of ‘1 M ZnSO_4_…’ as intended. A correct version of Fig. 5 is displayed below; the original caption remains unchanged.

**Fig. 5:**
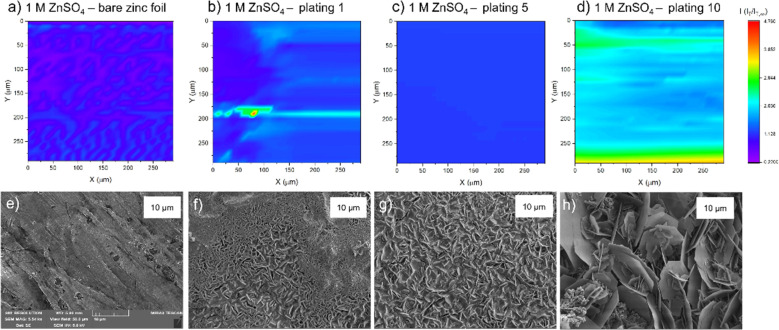
Scanning electrochemical microscope images of the same region of a zinc metal anode upon different stages of cycling at 1.7 mA h cm^−2^ in 1 M ZnSO_4_. (a) Bare zinc electrode. (b) After the first plating. (c) After the fifth plating. (d) After the tenth plating. SEM image of (e) zinc electrode soaked in electrolyte for one hour, (f) after the first plating, (g) after the fifth plating, (h) after the tenth plating. SEM images were taken with an excitation voltage of 5 kV in resolution mode with a view field of 50 μm.

The Royal Society of Chemistry apologises for these errors and any consequent inconvenience to authors and readers.

