# Supplementary material for: The effect of interface heterogeneity on zinc metal anode cyclability
Source: J Mater Chem A Mater. 2024 Aug 14;12(37):24916–33. doi: 10.1039/d4ta03165b (PMC11342068; doi:10.1039/d4ta03165b)
Supplement: TA-012-D4TA03165B-s001 [file TA-012-D4TA03165B-s001.pdf]

Supplementary Information (SI) for Journal of Materials Chemistry A.
This journal is © The Royal Society of Chemistry 2024

The effect of interface heterogeneity on zinc metal anode cyclability

J.T. Simon[1,3], V. Šedajová[1], D. Tripathy[1], H.E. Smith[1], S.M. Clarke[1,2*], C.P. Grey[1,3*], S. Menkin[1,3*]
[1]Yusuf Hamied Department of Chemistry, University of Cambridge, Lensfield Road, Cambridge, CB2 1EW, UK.
[2]Institute for Energy and Environmental Flows, University of Cambridge, Madingley Road, Cambridge, CB3 0EZ, UK.
[3]The Faraday Institution, Quad One, Harwell Science and Innovation Campus, Didcot, UK, OX11 0RA
* Corresponding author

ESI

Contents

Scanning Electrochemical Microscopy (SECM) 2
2 M $ZnSO_4$: 4
1 M $ZnSO_4$: 8
Galvanostatic cycling and impedance spectroscopy measurements. 13
XPS Analysis 24
Raman Analysis 25
SEM Images 28
References 32

Scanning Electrochemical Microscopy (SECM)

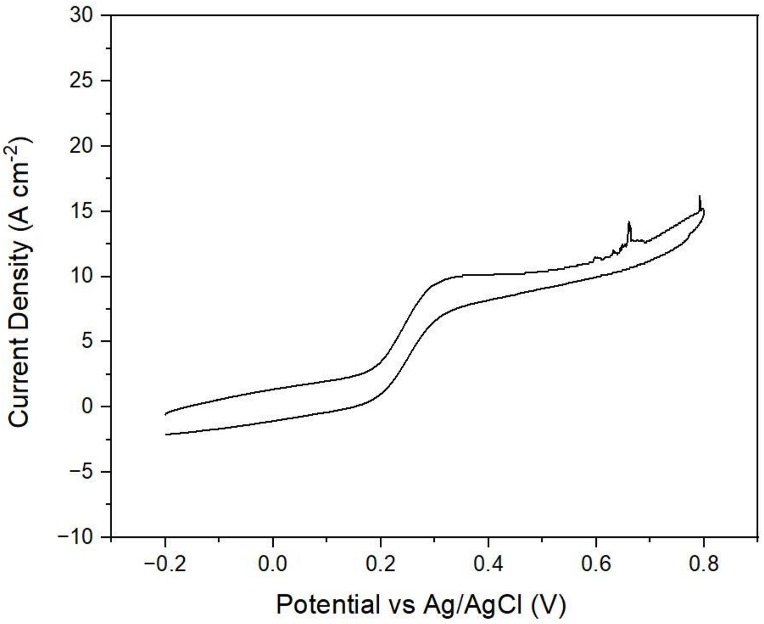

Figure S1: Cyclic voltammogram of 1 mM ferrocenemethanol in 0.01 M ZnSO4 at a 10 µm Pt ultramicroelectrode. Measurement was recorded at 30 mV s^{-1}.

Ferrocenemethanol was chosen as a redox mediator for this system because of its stability in aqueous solvents.[1] Figure S1 shows a cyclic voltammogram (CV) of 1 mM FcMeOH in 0.01 M $ZnSO_4$. At an ultramicroelectrode (UME), a recorded CV has a sigmoid-type shape, rather than the classic 'duck shape' recorded at larger electrodes. At large disk electrodes, mass transport towards the surface mostly occurs perpendicular to the surface, which is known as planar diffusion. Thus, as the voltage is increased or decreased, a diffusion limitation is reached, causing the current to decrease, and the usual 'duck shape' CV to be recorded. Mass transport to UMEs occurs by hemispherical diffusion, meaning that diffusion of redox species to the surface comes from all directions. Consequently, no mass transport limitation is seen unless extremely high sweep rates are used. Thus, a sigmoidal steady-state voltammogram is recorded, where the plateaus seen are the kinetic limitation of electron transfer.[2] For the FcMeoH/$FcMeOH^+$ redox couple, a probe voltage of +0.5 V vs Ag/AgCl was used to drive the oxidation of the mediator.

The voltage traces of zinc plating and stripping prior to the SECM measurements are depicted in Figures S2-S21. In a symmetric coin cell, the voltage trace reflects the sum of the response of both electrodes. In the SECM, when the WE is plated, the CE is stripped, and vice versa; however, it is expected that the electrolysis processes will be more dominant on the counter

electrode since the counter electrode (CE - Pt) is prone to hydrogen evolution. Hence, the voltage trace mainly reflects the stripping process on the WE.

The plating voltage traces (a) typically show a decrease in the potential and then a plateau at approximately -0.9V vs. Ag/AgCl. The initial drop is due to the nucleation overpotential that adds to the reduction potential and the overpotential contributed by the bulk resistance of the electrolyte. Since the time interval between the collected points is longer than the peak time, the nucleation peaks were not recorded.

(b) As expected, no nucleation peaks were recorded during stripping since we estimate that zinc plating on the Pt-CE was minimal. The slight overpotential decrease during the stripping is attributable to the reduction of the porosity of the electrode.

The overpotential for the processes in 1 M ZnSO4 is slightly higher than in 2 M; this could be due to the higher conductivity of the 2 M electrolyte and the lower zinc reduction potential in the more concentrated electrolyte.

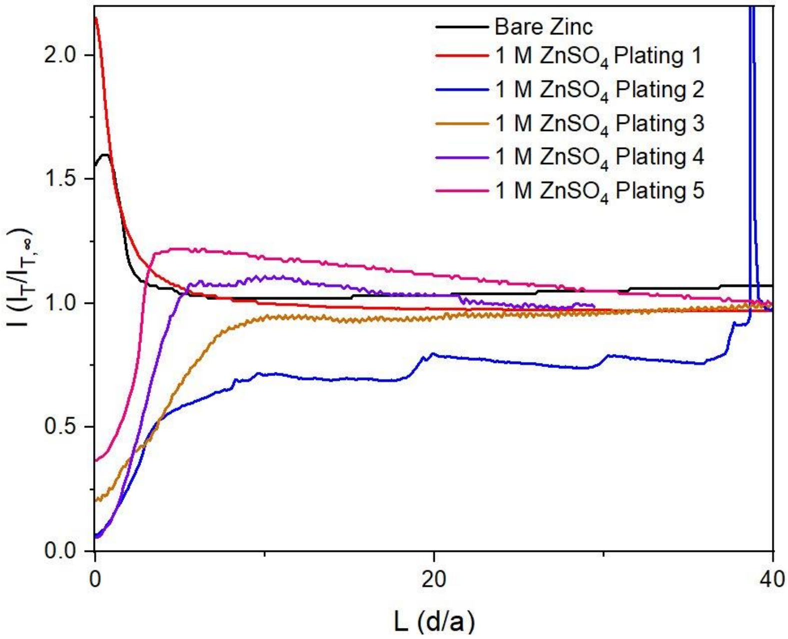

Figure S2: Repeat data for probe approach curves for plating one through five.

Probe approach curve measurements were repeated, including measurements for plating 2-5 to investigate how the kinetics of electron transfer change in the initial cycling of the cell. Positive feedback was observed for both bare zinc and plating 1, and the feedback response transitioned to negative feedback for plating 2-5. This indicates that the interface becomes more insulating following the first plating.

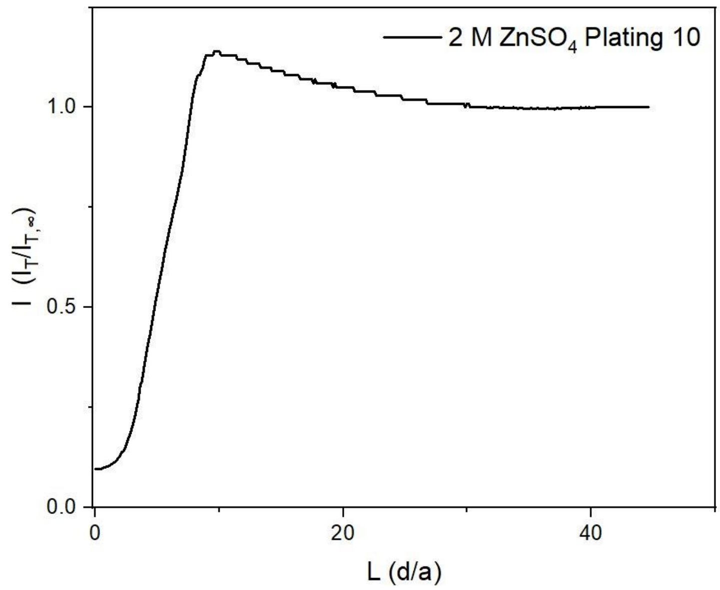

Figure S3: Repeat probe approach curve measurement for 2 M $ZnSO_4$ plating ten.

2 M $ZnSO_4$:

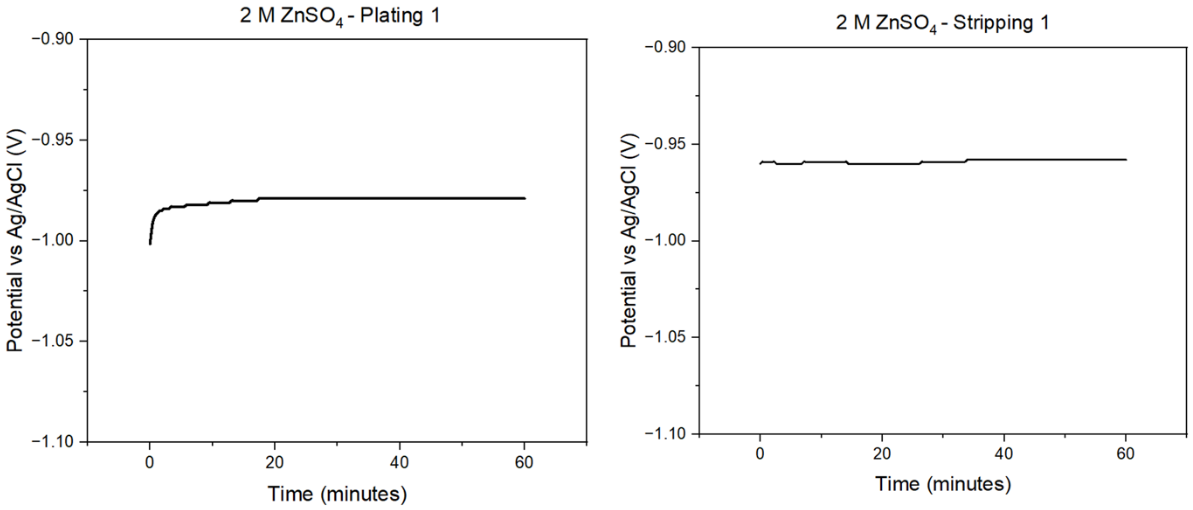

Figure S4: Zinc electroplating cycle 1: a) Plating at 1.7 mAh cm^{-2}. b) Stripping at 1.7 mAh cm^{-2}.

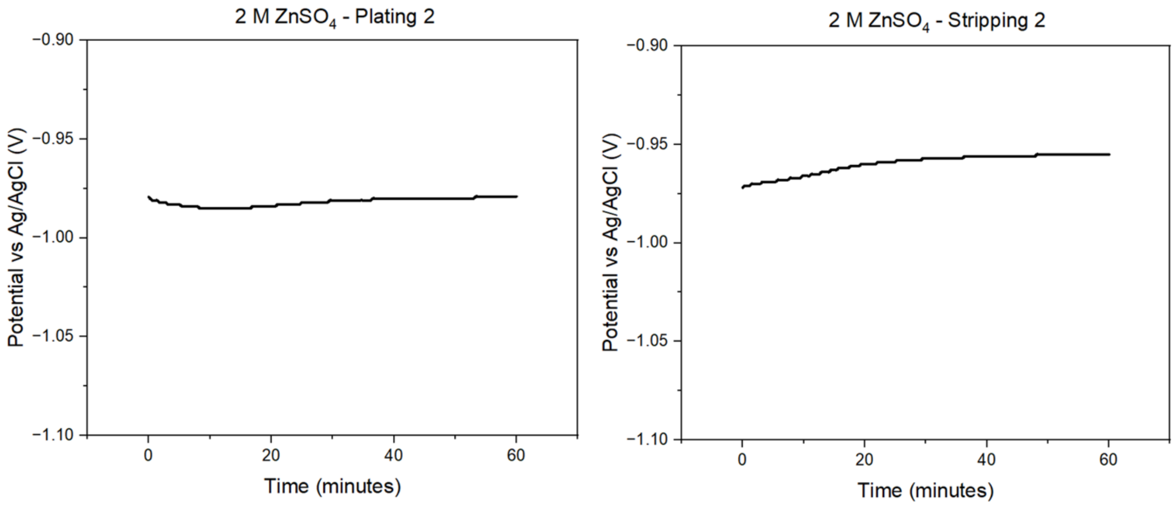

Figure S5: Zinc electroplating cycle 2: a) Plating at 1.7 mAh cm^{-2}. b) Stripping at 1.7 mAh cm^{-2}.

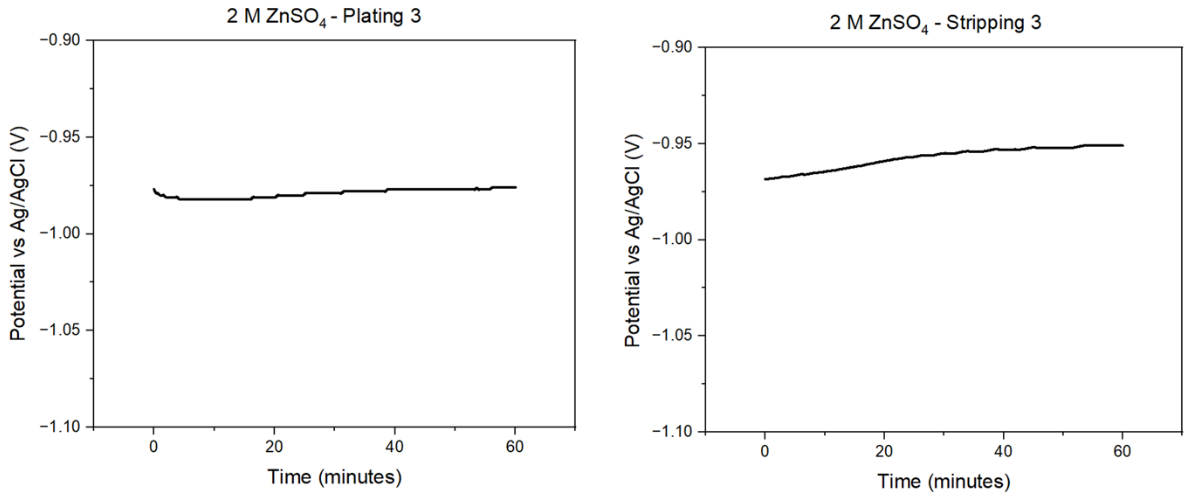

Figure S6: Zinc electroplating cycle 3: a) Plating at 1.7 mAh cm^{-2}. b) Stripping at 1.7 mAh cm^{-2}

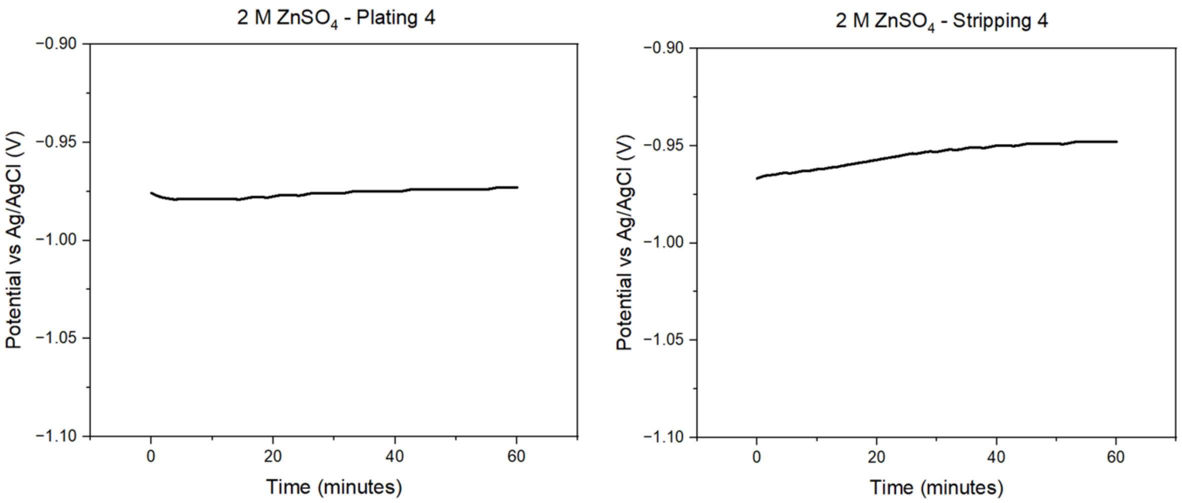

Figure S7: Zinc electroplating cycle 4: a) Plating at 1.7 mAh cm^{-2}. b) Stripping at 1.7 mAh cm^{-2}.

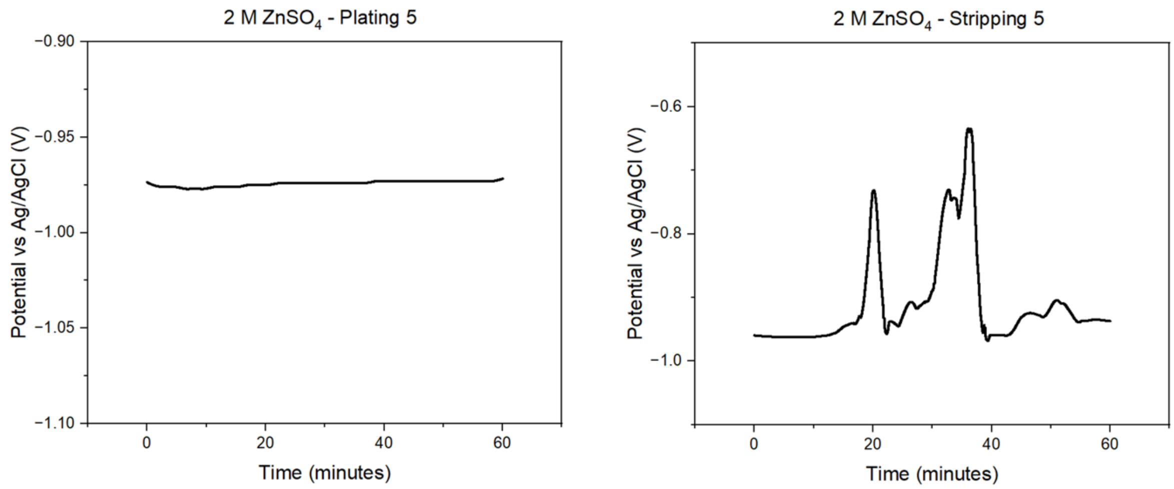

Figure S8: Zinc electroplating cycle 5: a) Plating at 1.7 mAh cm^{-2}. b) Stripping at 1.7 mAh cm^{-2}.

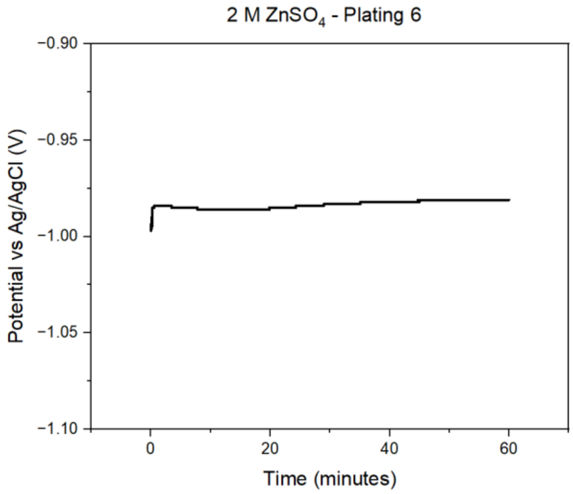

Figure S9: Zinc electroplating cycle 6: Plating at 1.7 mAh cm^{-2}.

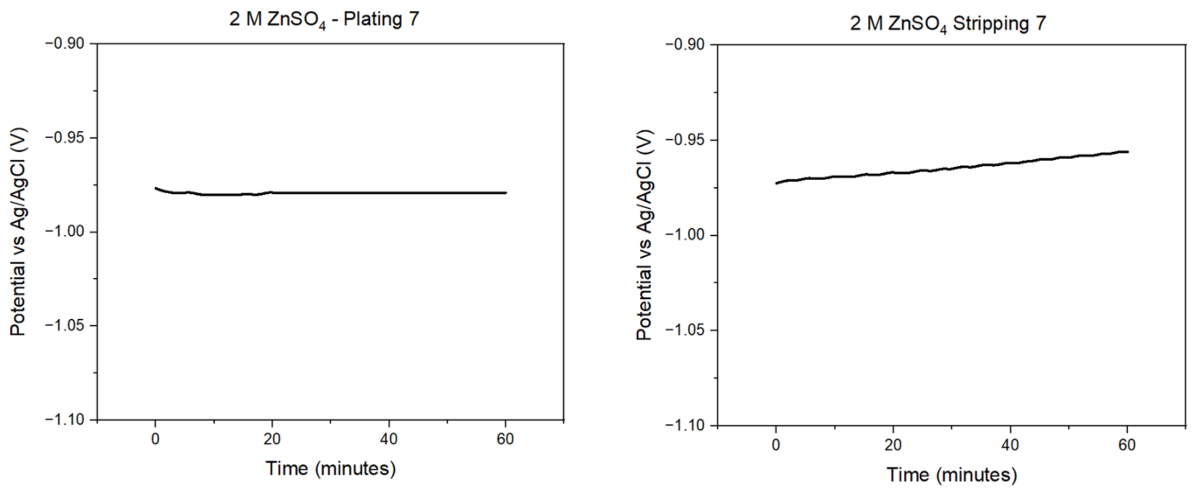

Figure S10: Zinc electroplating cycle 7: a) Plating at 1.7 mAh cm^{-2}. b) Stripping at 1.7 mAh cm^{-2}

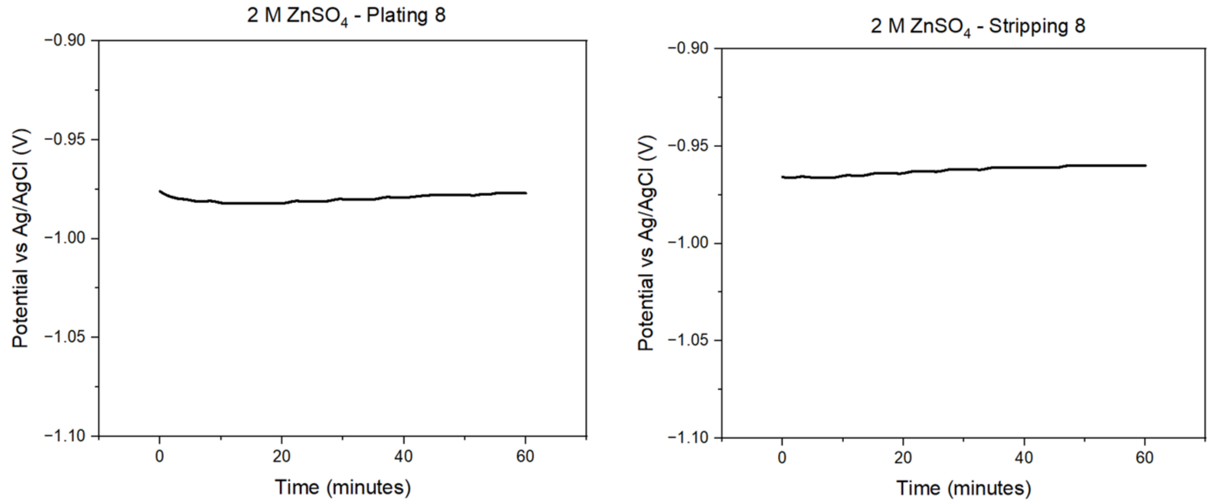

Figure S11: Zinc electroplating cycle 8: a) Plating at 1.7 mAh cm^{-2}. b) Stripping at 1.7 mAh cm^{-2}.

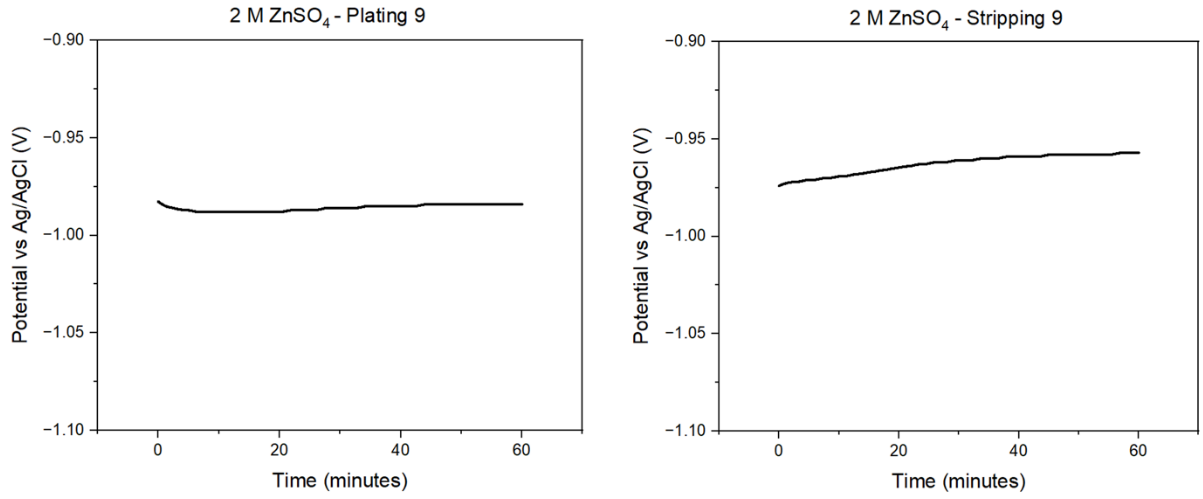

Figure S12: Zinc electroplating cycle 9: a) Plating at 1.7 mAh cm^{-2}. b) Stripping at 1.7 mAh cm^{-2}

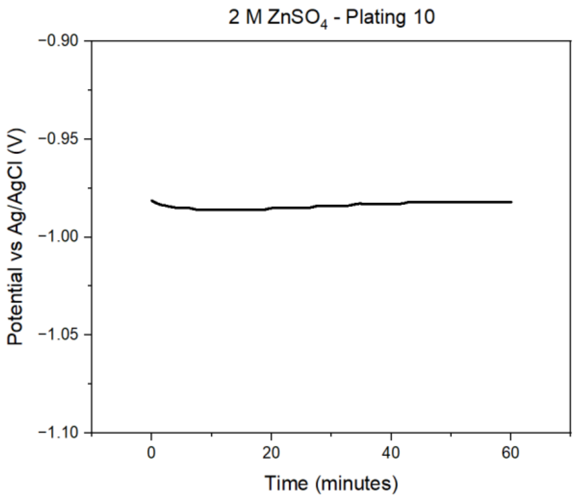

Figure S13: Zinc electroplating cycle 10: Plating at 1.7 mAh cm^{-2}.

1 M $ZnSO_4$:

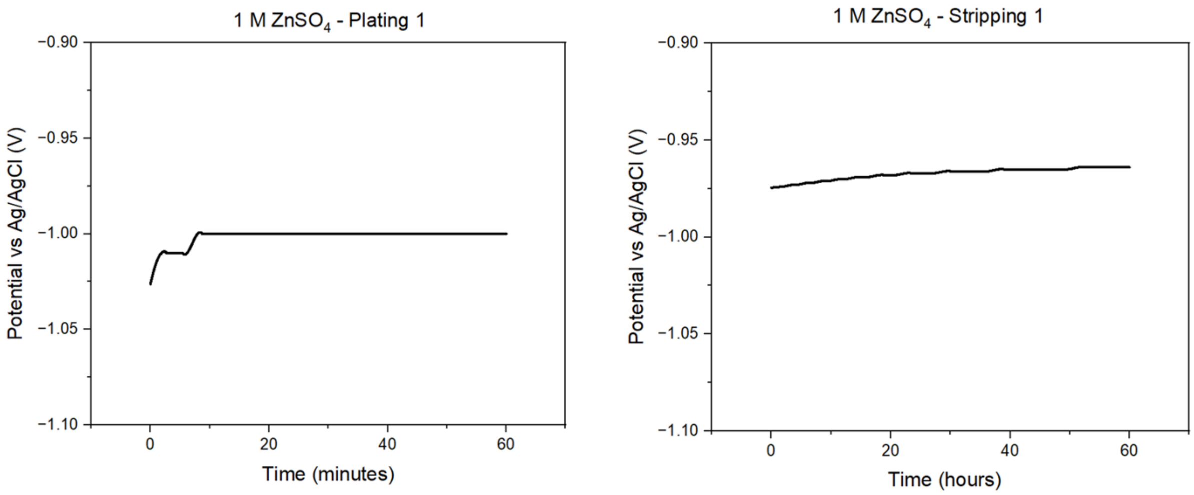

Figure S14: Zinc electroplating cycle 1: a) Plating at 1.7 mAh cm^{-2}. b) Stripping at 1.7 mAh cm^{-2}.

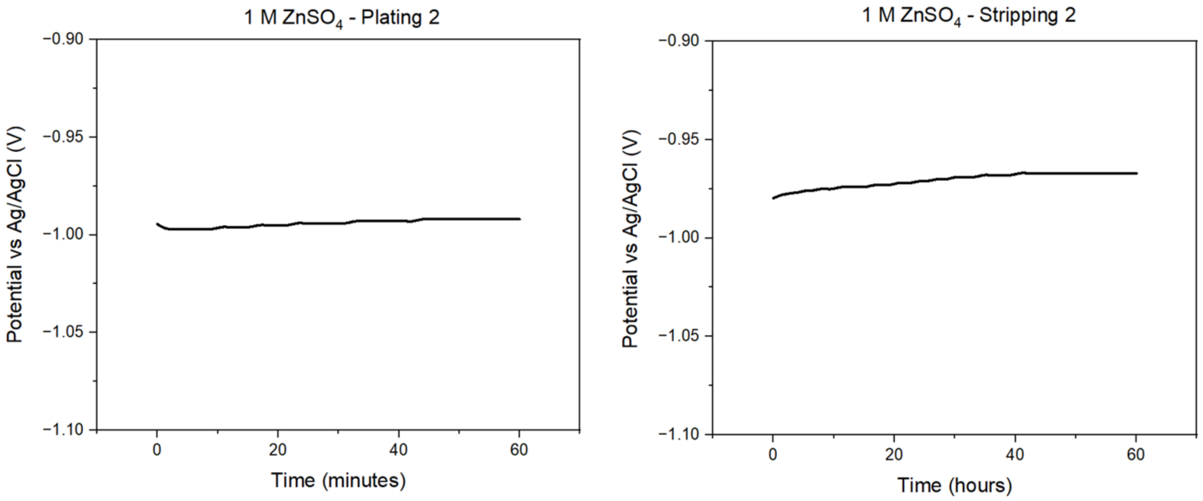

Figure S15: Zinc electroplating cycle 2: a) Plating at 1.7 mAh cm^{-2}. b) Stripping at 1.7 mAh cm^{-2}.

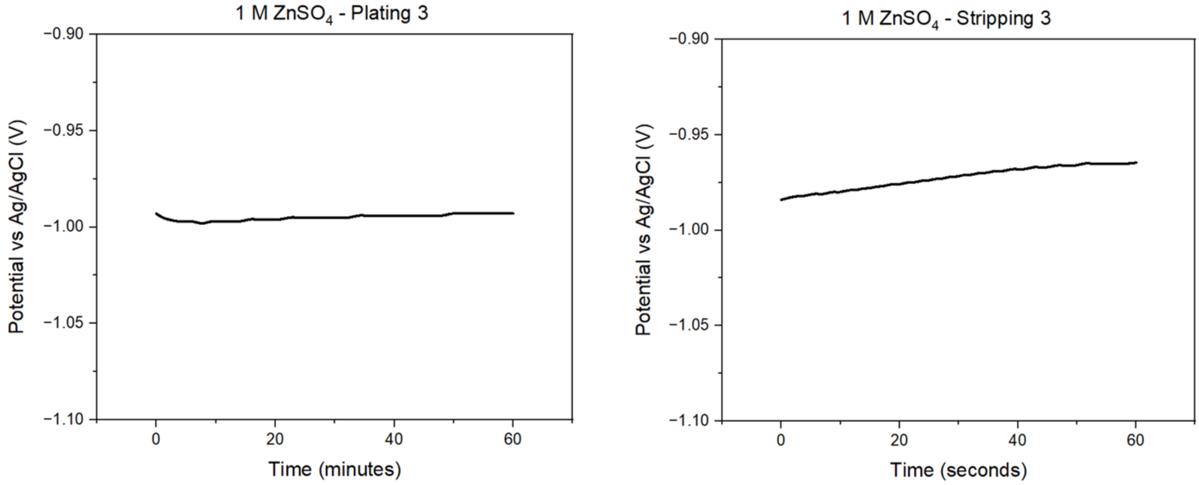

Figure S16: Zinc electroplating cycle 3: a) Plating at 1.7 mAh cm^{-2}. b) Stripping at 1.7 mAh cm^{-2}.

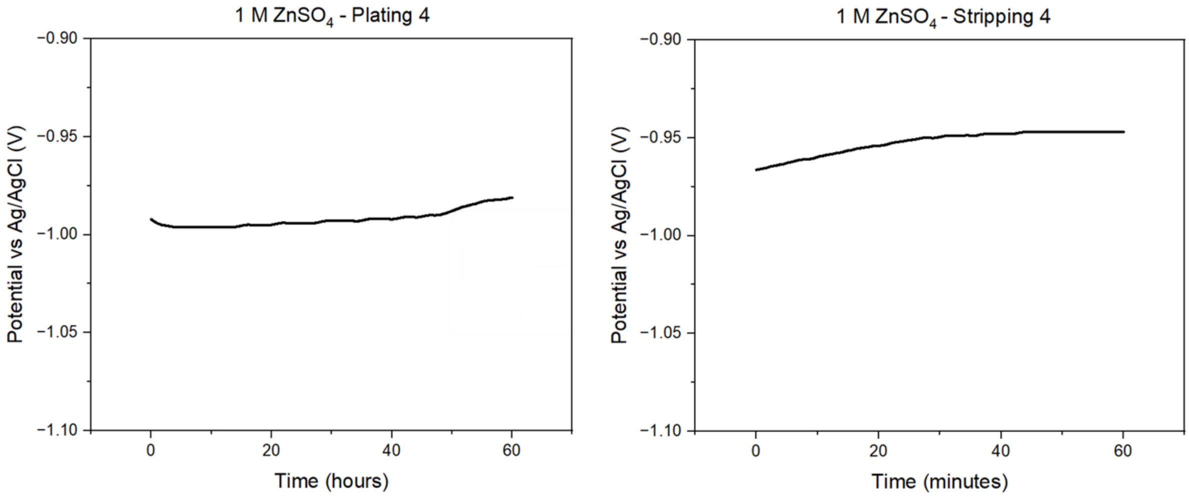

Figure S17: Zinc electroplating cycle 4: a) Plating at 1.7 mAh cm^{-2}. b) Stripping at 1.7 mAh cm^{-2}.

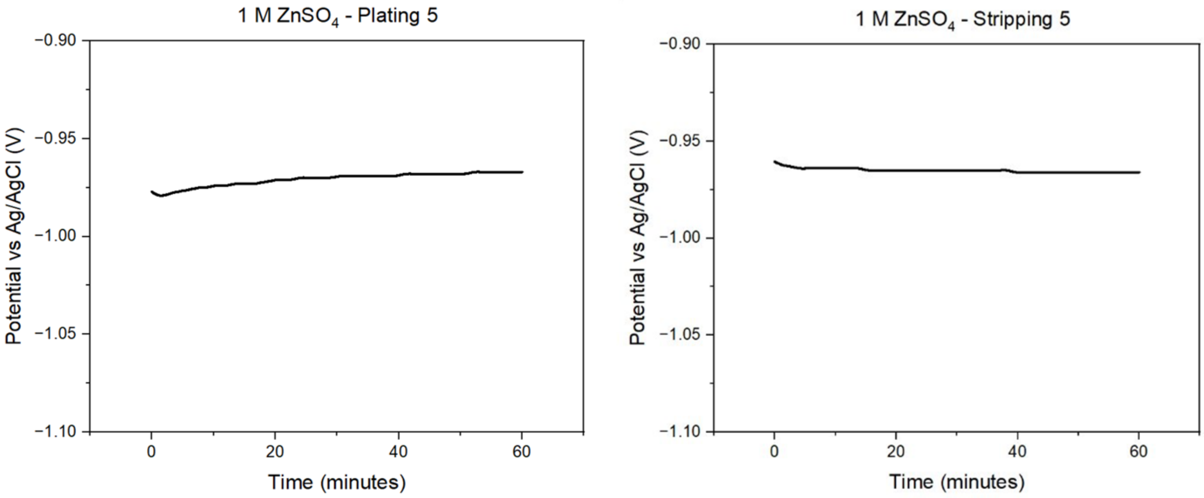

Figure S18: Zinc electroplating cycle 4: a) Plating at 1.7 mAh cm^{-2}. b) Stripping at 1.7 mAh cm^{-2}.

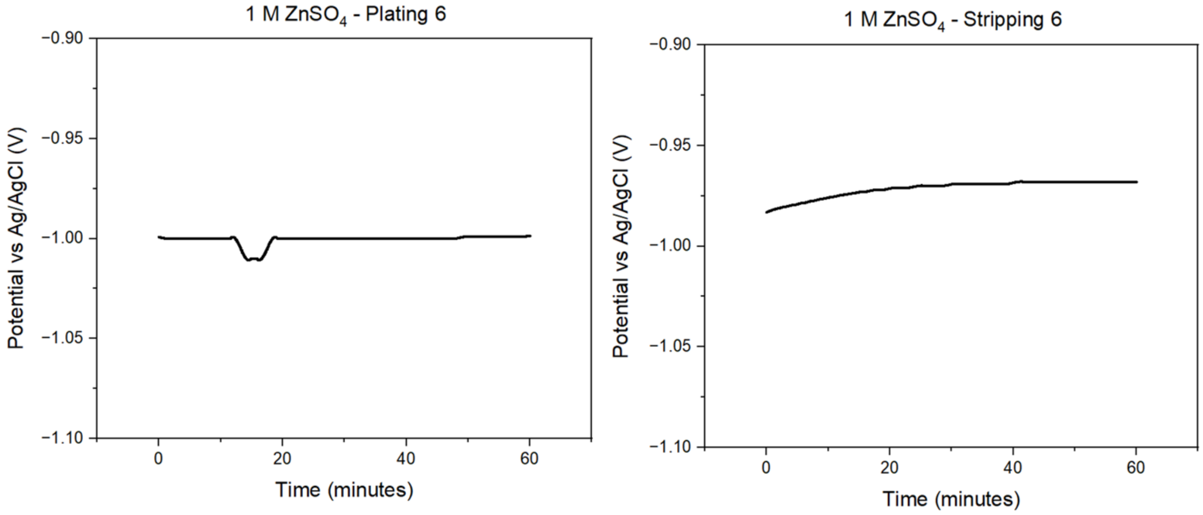

Figure S19: Zinc electroplating cycle 6: a) Plating at 1.7 mAh cm^{-2}. b) Stripping at 1.7 mAh cm^{-2}.

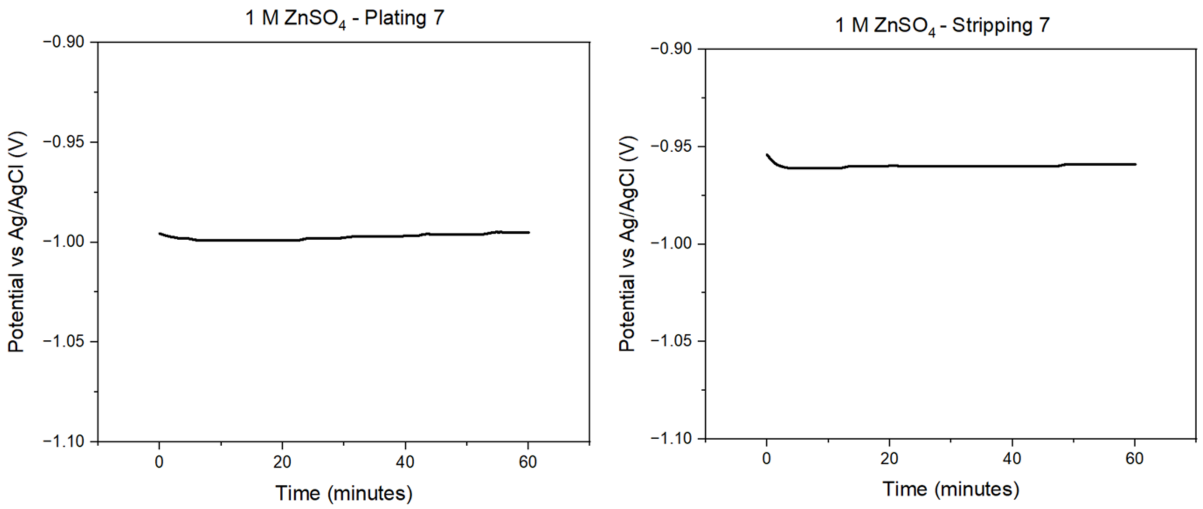

Figure S20: Zinc electroplating cycle 7: a) Plating at 1.7 mAh cm^{-2}. b) Stripping at 1.7 mAh cm^{-2}.

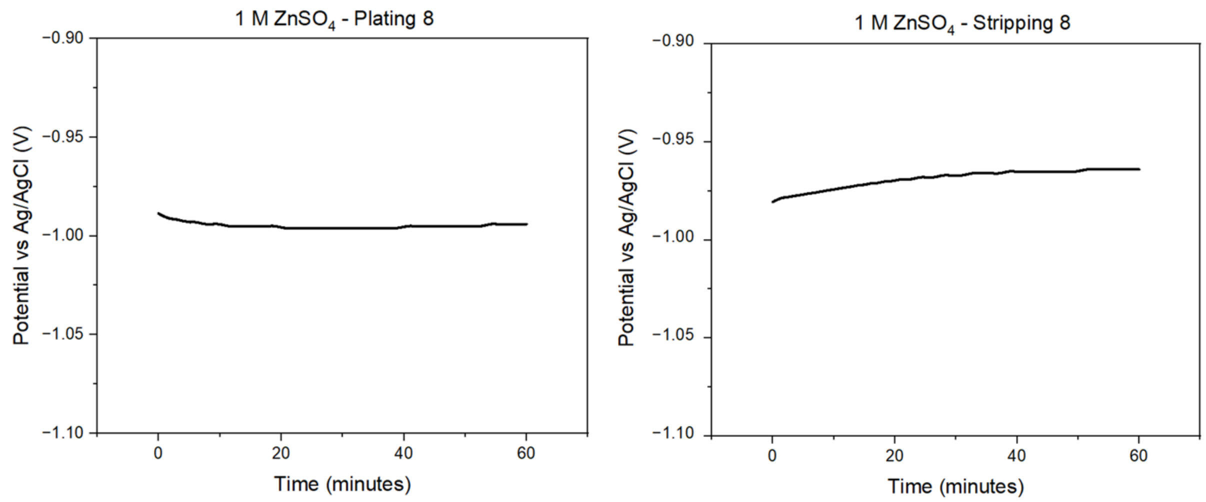

Figure S21: Zinc electroplating cycle 8: a) Plating at 1.7 mAh cm^{-2}. b) Stripping at 1.7 mAh cm^{-2}.

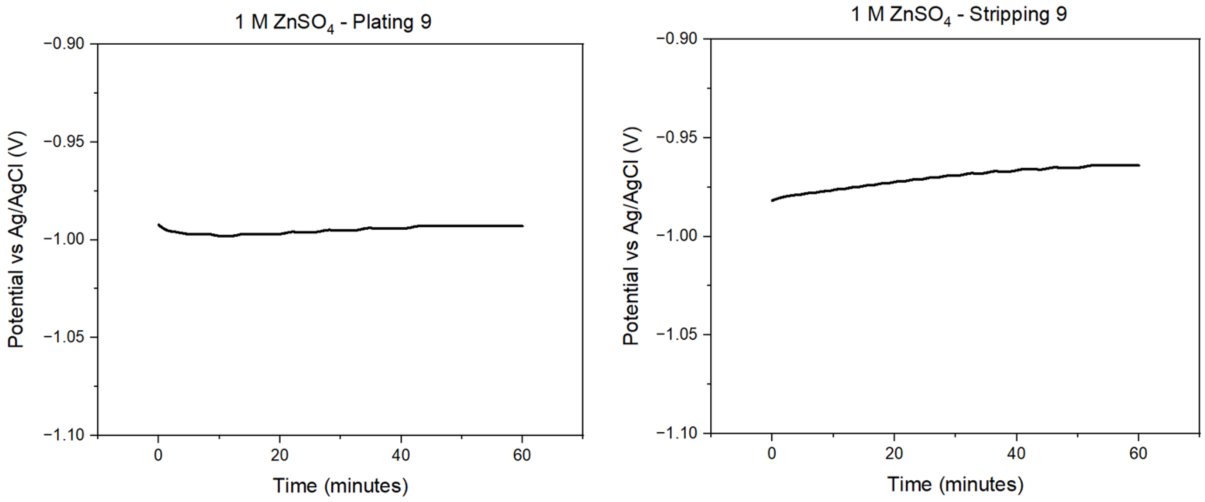

Figure S22: Zinc electroplating cycle 9: a) Plating at 1.7 mAh cm^{-2}. b) Stripping at 1.7 mAh cm^{-2}.

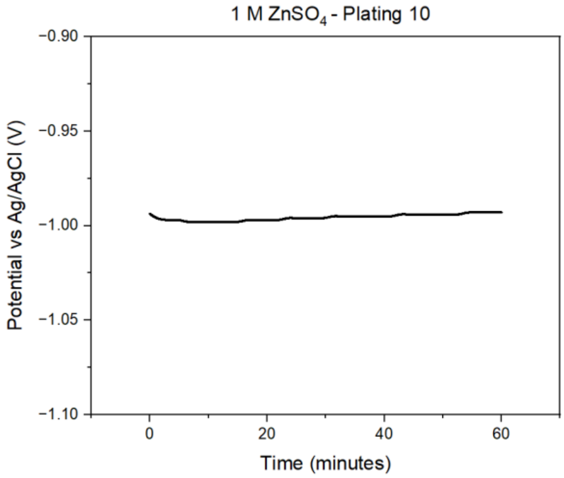

Figure S23: Zinc electroplating cycle 10: Plating at 1.7 mAh cm^{-2}.

Galvanostatic cycling and impedance spectroscopy measurements.

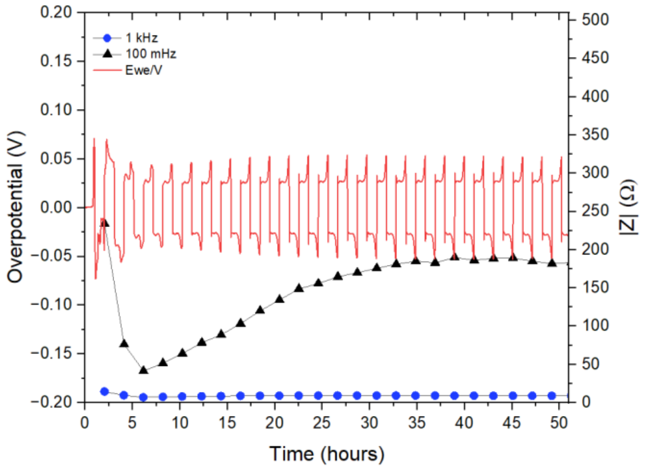

Figure S24*:* Left: A Voltage trace (E vs. t) of successive galvanostatic cycles at 0.5 mA h cm^{-2} of zinc in a symmetric cell with 2 M $ZnSO_4$ aqueous electrolyte and a GF separator. This is equivalent to constant current density 0.5mA cm^{-2} at 1 C (plating and stripping 0.5mAh cm^{-2}, which is equivalent to 1.2 mg cm^{-2} zinc). Right: impedance modulus at 0.1Hz (black), 1kHz (blue).

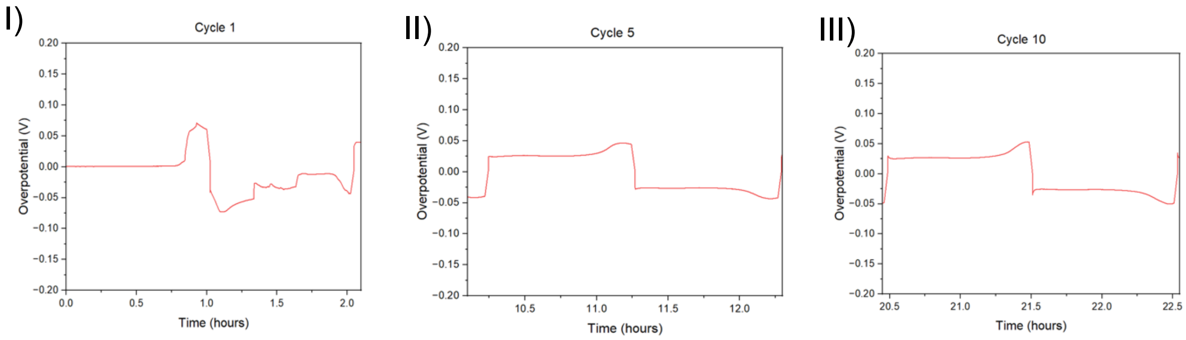

Figure S25: Magnification of selected voltage traces (E vs. t) of successive galvanostatic cycling of a total of 0.5 mAh cm^{-2} of Zn in 2 M $ZnSO_4$ aqueous electrolyte. Cycles 1 (I),5 (II), and 10 (III) are presented.

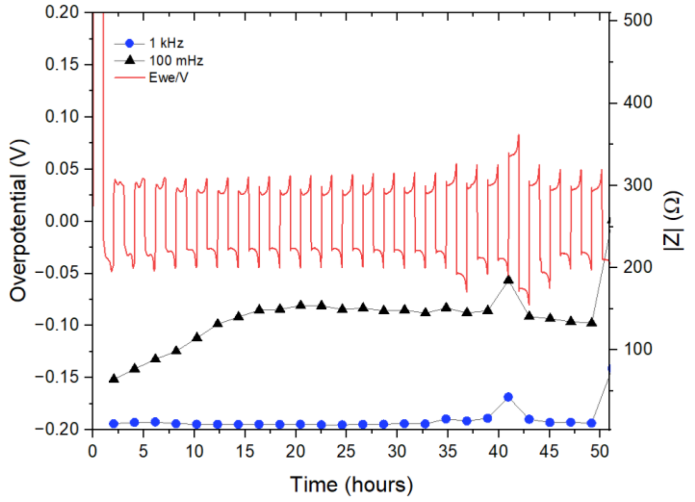

Figure S26: A Voltage trace (E vs. t) of successive galvanostatic cycling at 0.5 mA h cm^{-2} of zinc in a symmetric cell with 1 M $ZnSO_4$ aqueous electrolyte and a GF separator. Constant current density 0.5mA cm^{-2} at 1 C (plating and stripping 0.5mAh cm^{-2}, which is equivalent to 1.2 mg cm^{-2} zinc). Right: impedance modulus at 0.1Hz (black), 1kHz (blue).

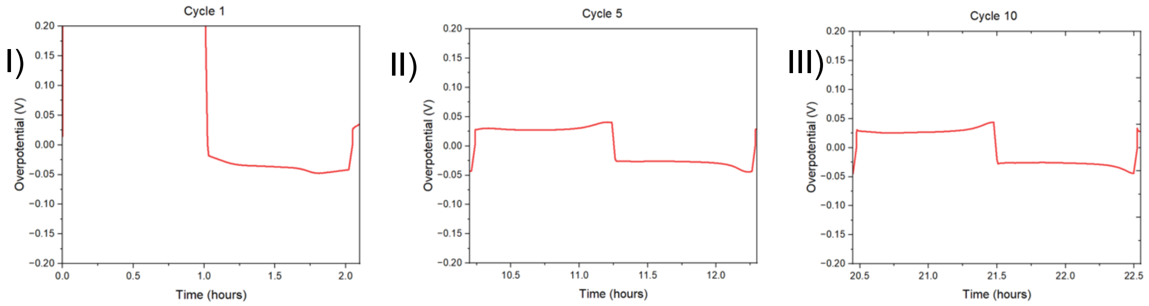

Figure S27*:* Magnification of selected voltage traces (E vs. t) of successive galvanostatic cycling of a total of 0.5 mAh cm^{-2} of Zn in 2 M $ZnSO_4$ aqueous electrolyte. Cycles 1 (I),5 (II), and 10 (III) are presented

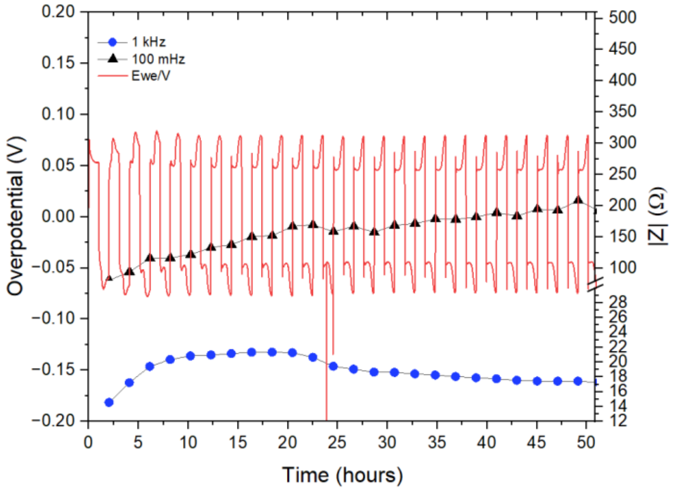

Figure S28: Left: A Voltage trace (E vs. t) of successive galvanostatic cycling at 0.5 mA h cm^{-2} of zinc in a symmetric cell with 2 M $ZnSO_4$ aqueous electrolyte and a Celgard 3501 polymer separator. Constant current density 0.5mA cm^{-2} at 1 C (plating and stripping 0.5mAh cm^{-2}, which is equivalent to 1.2 mg cm^{-2} zinc).). Right: impedance modulus at 0.1Hz (black), 1kHz (blue). Repeat Data.

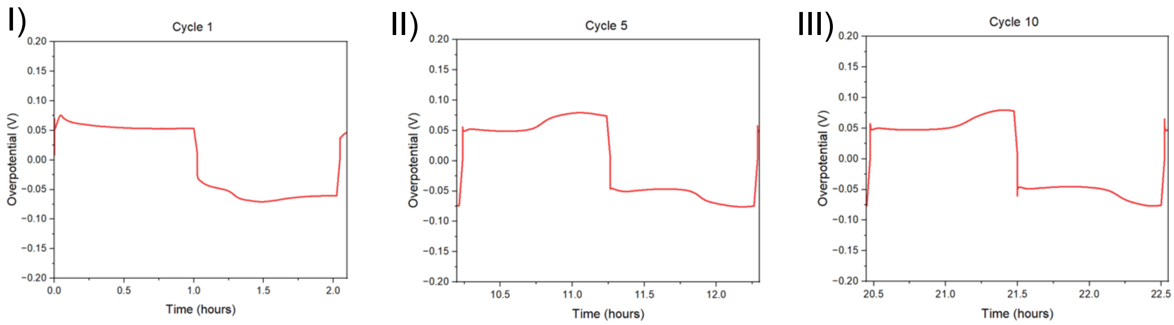

Figure S29: Magnification of selected voltage traces (E vs. t) of successive galvanostatic cycling at 0.5 mAh cm^{-2} of zinc in 2 M $ZnSO_4$. Cycles 1 (I),5 (II), and 10 (III) are presented.

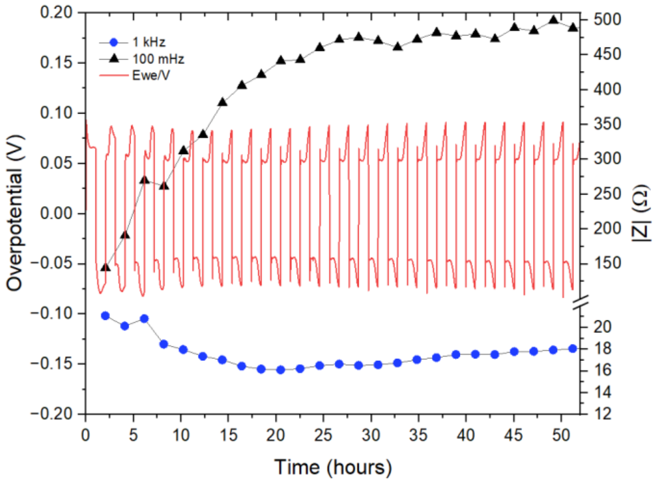

Figure S30: Left: A Voltage trace (E vs. t) of successive galvanostatic cycling at 0.5 mA h cm^{-2} of zinc in a symmetric cell with 1 M $ZnSO_4$ aqueous electrolyte and a Celgard 3501 polymer separator. Constant current density 0.5mA cm^{-2} at 1 C (plating and stripping 0.5mAh cm^{-2}, which is equivalent to 1.2 mg cm^{-2} zinc).). Right: impedance modulus at 0.1Hz (black), 1kHz (blue). Repeat Data.

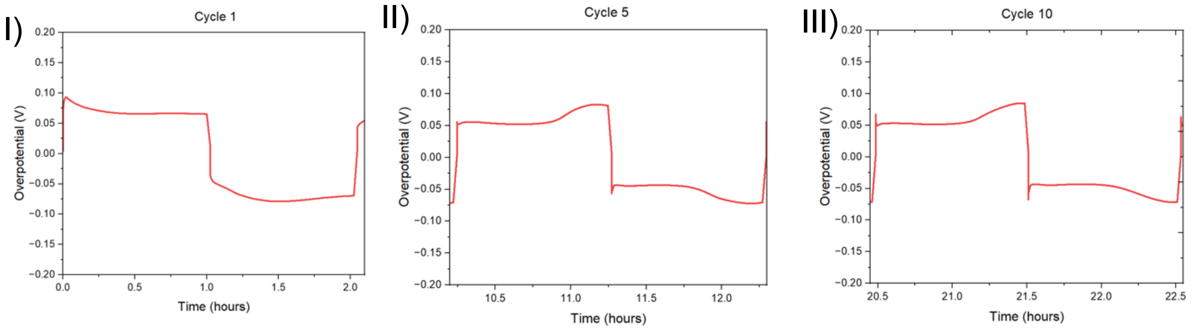

Figure S31: Magnification of selected voltage traces (E vs. t) of successive galvanostatic cycling at 0.5 mAh cm^{-2} of zinc in 1 M $ZnSO_4$. Cycles 1 (I),5 (II), and 10 (III) are presented.

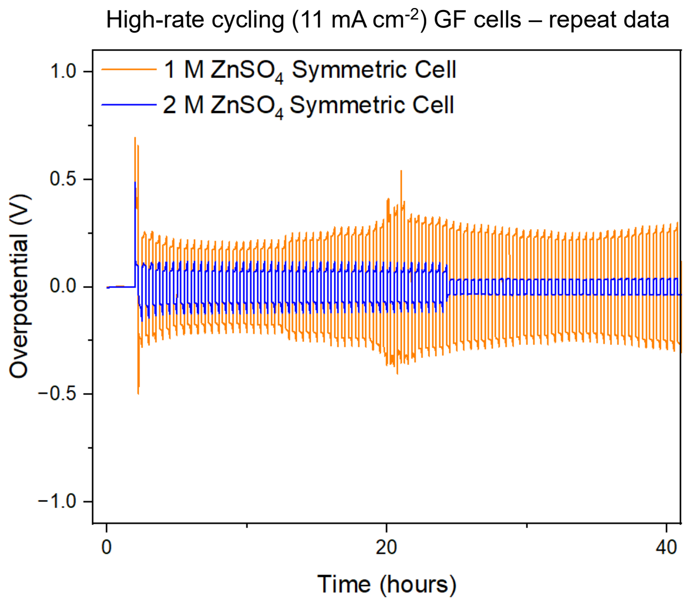

Figure S32: A Voltage trace (E vs. t) of successive galvanostatic cycling at 11mA cm^{-2} of 0.5 mAh cm^{-2} Zn in Zn symmetric cell with 1 M and 2 M $ZnSO_4$ aqueous electrolytes using a GF separator.

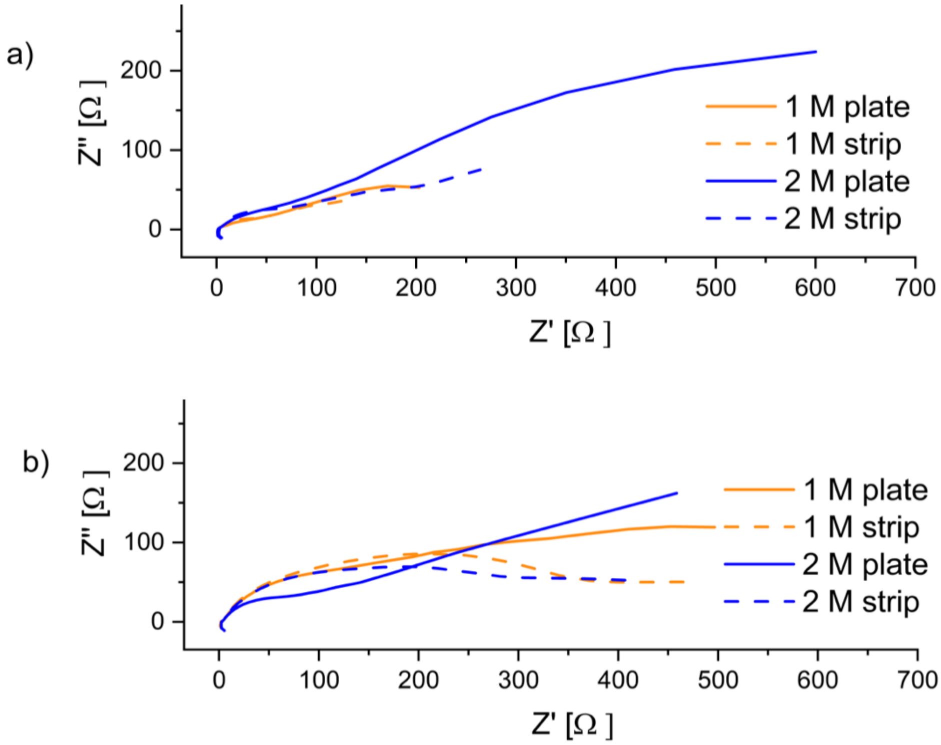

Figure S33: PEIS Nyquist plots, amplitude 5 mV, 100 kHz - 1 Hz, in symmetric Zn cells with 1-2 M ZnSO4 electrolyte and a polymer (Celgard 3501) separator, plating and stripping 0.5 mAh cm−2 Zn. PEIS measured after (a) a single cycle (b) after 15 plating.

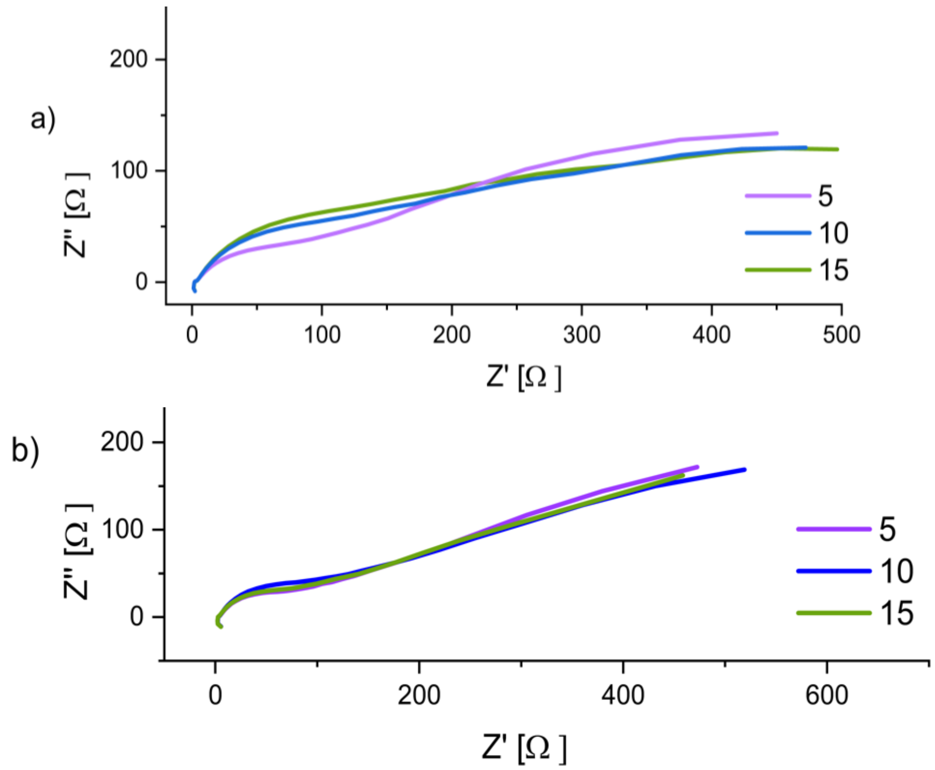

Figure S34: PEIS Nyquist plots, amplitude 5 mV, 100 kHz - 1 Hz, in symmetric Zn cells and a polymer (Celgard 3501) separator, plating and stripping 0.5 mAh cm^{-2} Zn. PEIS measured after 5,10 and 15 cycles (a) in 1 M and (b) 2 M $ZnSO_4$ electrolyte.

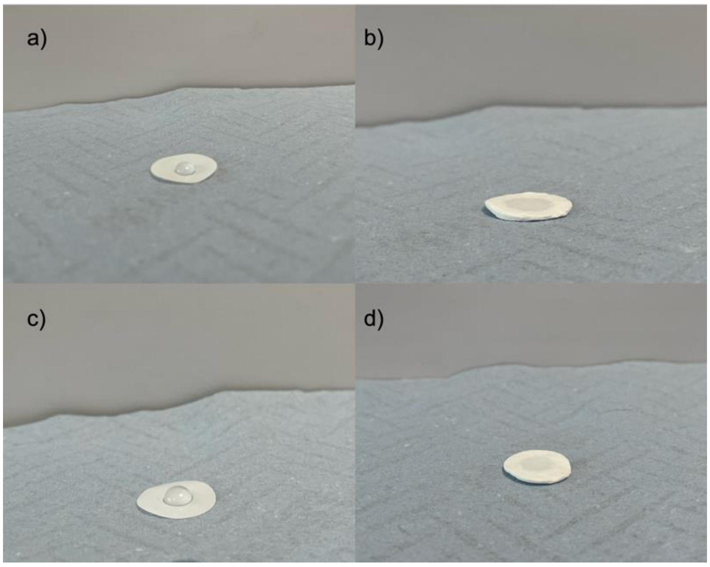

Figure S35: (a) 2 M $ZnSO_4$ on a Celgard 3501 separator. (b) 2 M $ZnSO_4$ on a GF separator. (c) 1 M $ZnSO_4$ on a Celgard 3501 separator. (d) 1 M $ZnSO_4$ on a GF separator.

An experimental test whereby a drop of electrolyte was added to both Celgard 3501 (polymer) and GF separators was performed. Both electrolytes formed a drop on top of the polymer

separator, showing the hydrophobic nature. Comparatively, both electrolytes soaked into the GF separator, demonstrating the hydrophilic nature.

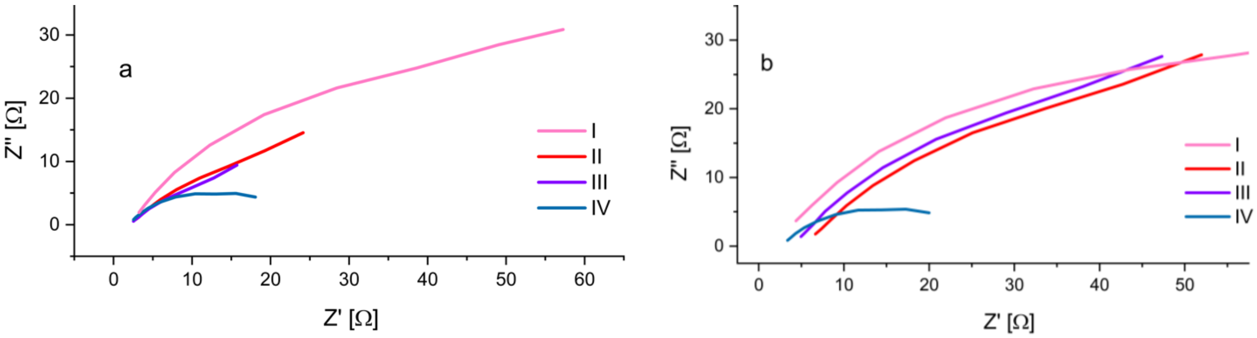

Figure S36: GEIS Nyquist plots, amplitude 100 µA, 500 Hz - 1 Hz, in symmetric Zn cell with ZnSO4 electrolyte and a GF separator at 10°C. Cycle 1: before cycling (I), after plating 1.25 mAh cm−2 Zn on the cathode (II), after plating 2.5 mAh cm−2 Zn on the cathode (III), after stripping 1.25 mAh cm−2 Zn from the cathode (IV). (a) 1 M (b) 2 M.

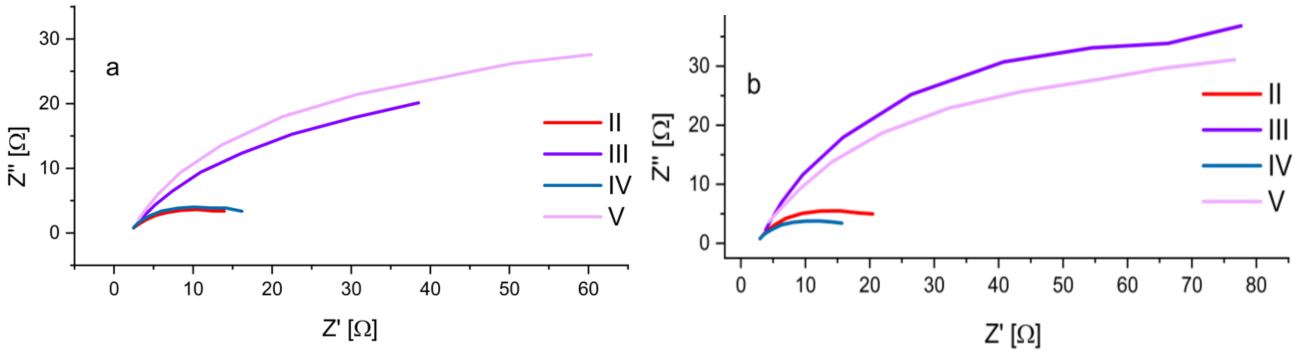

Figure S37: GEIS Nyquist plots, amplitude 100 µA, 500 Hz - 1 Hz, in symmetric Zn cell with ZnSO4 electrolyte and a GF separator at 10°C. Cycle 4 : after plating 1.25 mAh cm−2 Zn on the cathode (II), after plating 2.5 mAh cm−2 Zn on the cathode (III), after stripping 1.25 mAh cm−2 Zn from the cathode (IV), after stripping 2.5 mAh cm−2 Zn from the cathode (IV). (a) 1 M (b) 2 M.

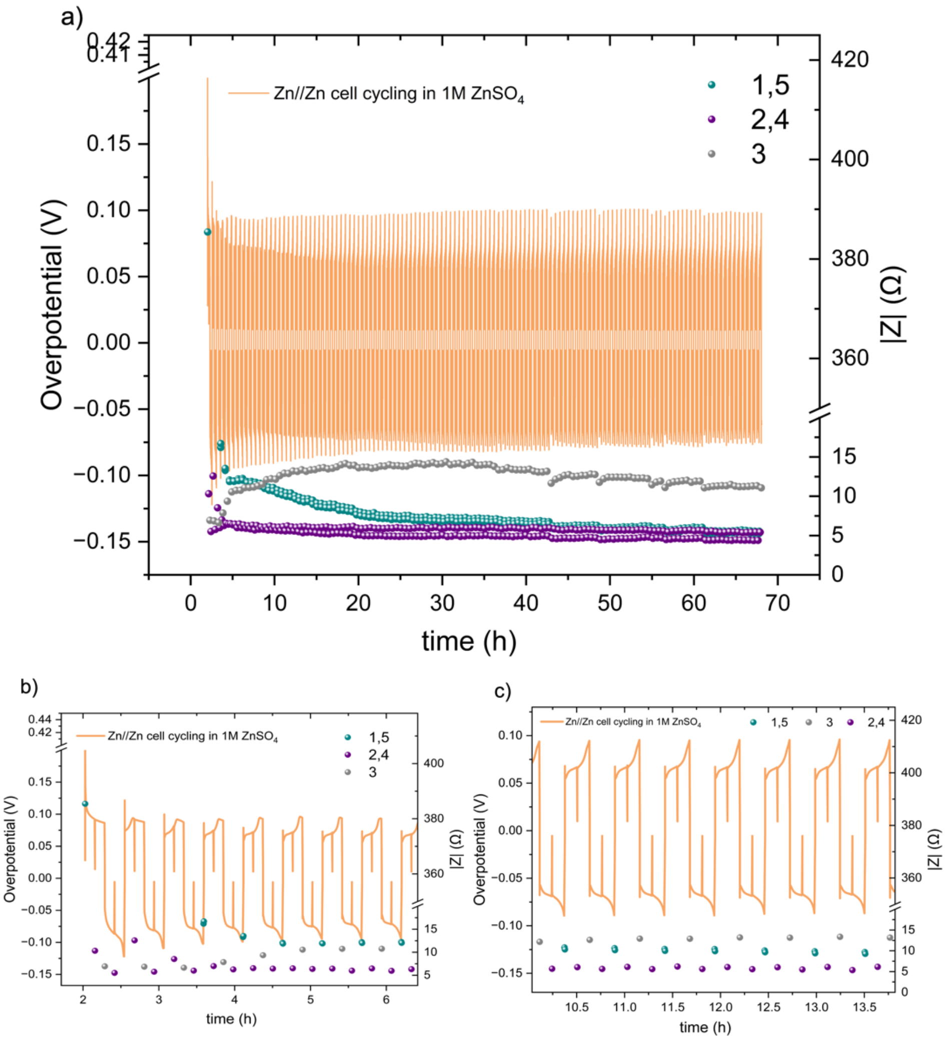

a)
Zn//Zn cell cycling in 1M $ZnSO_4$
1,5
2,4
3
Overpotential (V)
|Z| (Ω)
time (h)
b)
Zn//Zn cell cycling in 1M $ZnSO_4$
1,5
2,4
3
Overpotential (V)
|Z| (Ω)
time (h)
c)
Zn//Zn cell cycling in 1M $ZnSO_4$
1,5
3
2,4
Overpotential (V)
|Z| (Ω)
time (h)

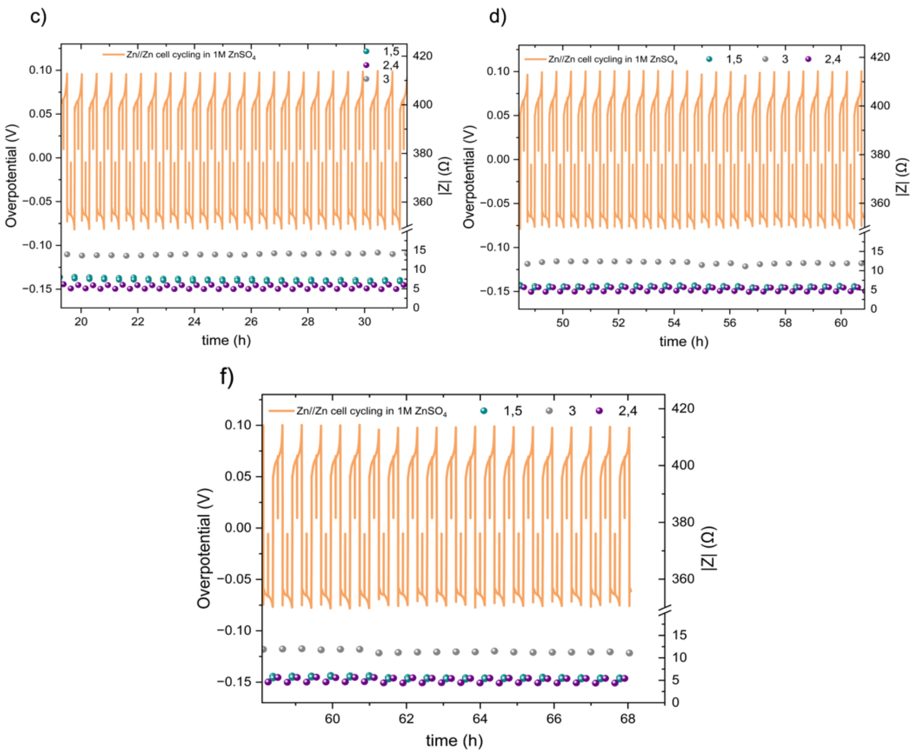

Figure S38: (a) An overpotential trace (overpotential vs t) of successive galvanostatic cycling of a total of 1.25 mAh cm-2 of Zn at 10 mA cm-2 in zinc symmetric cell with 1 M ZnSO4 aqueous electrolyte, GF separator, at 10 °C (left Y axe) and the total impedance at 10 Hz, measured during OCV (right Y axe). The galvanostatic impedance spectra (GEIS) were recorded every 1.25 hours, between 500 - 10 Hz with 100 µA amplitude. The single-spectrum measurement time was nine seconds. A drop in the overpotential (a spike in the overpotential trace) was observed when the GEIS measurements were taken at stages II and IV (b-f).

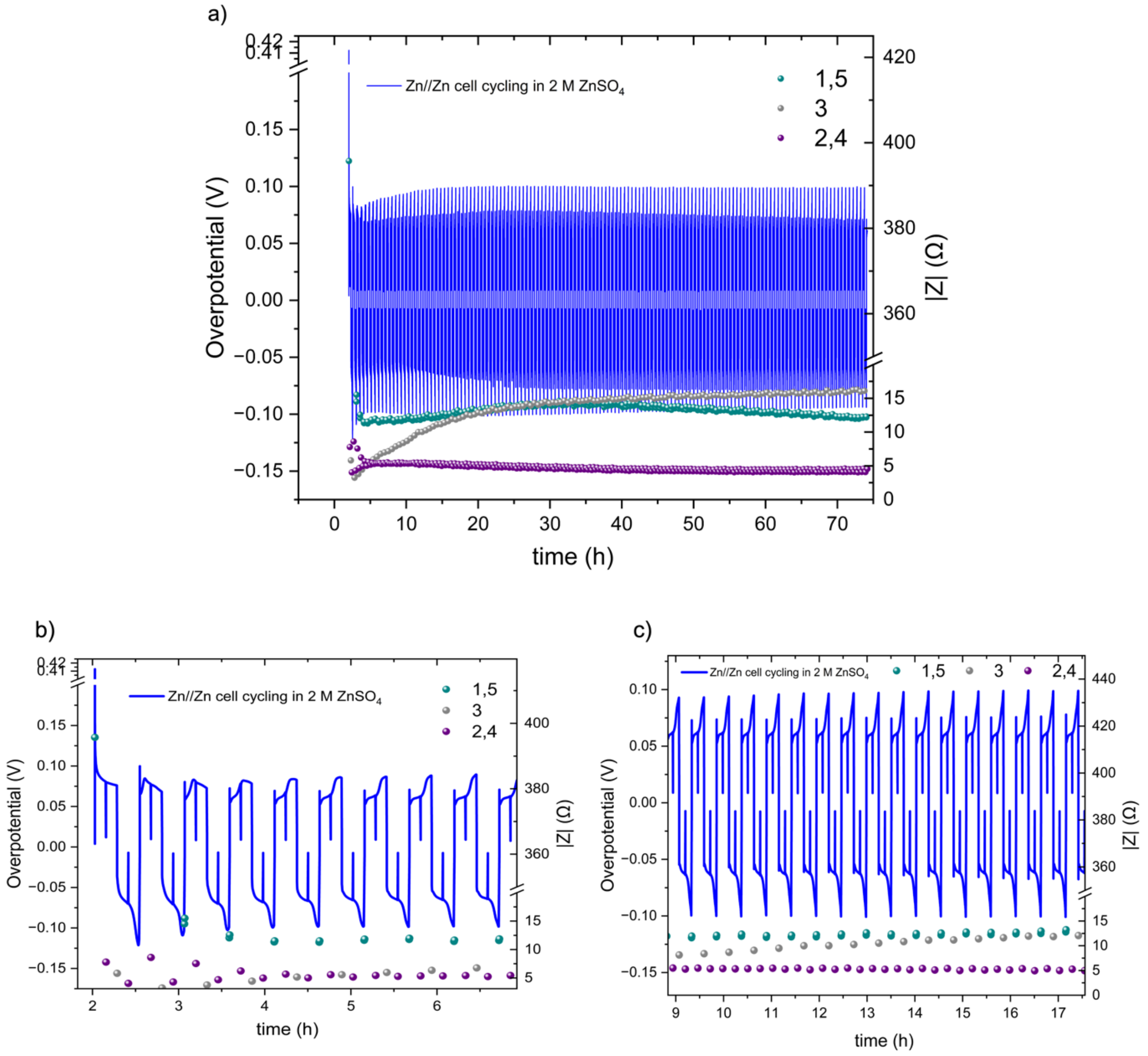

a)
Zn//Zn cell cycling in 2 M $ZnSO_4$
1,5
3
2,4
Overpotential (V)
|Z| (Ω)
time (h)
b)
Zn//Zn cell cycling in 2 M $ZnSO_4$
1,5
3
2,4
Overpotential (V)
|Z| (Ω)
time (h)
c)
Zn//Zn cell cycling in 2 M $ZnSO_4$
1,5
3
2,4
Overpotential (V)
|Z| (Ω)
time (h)

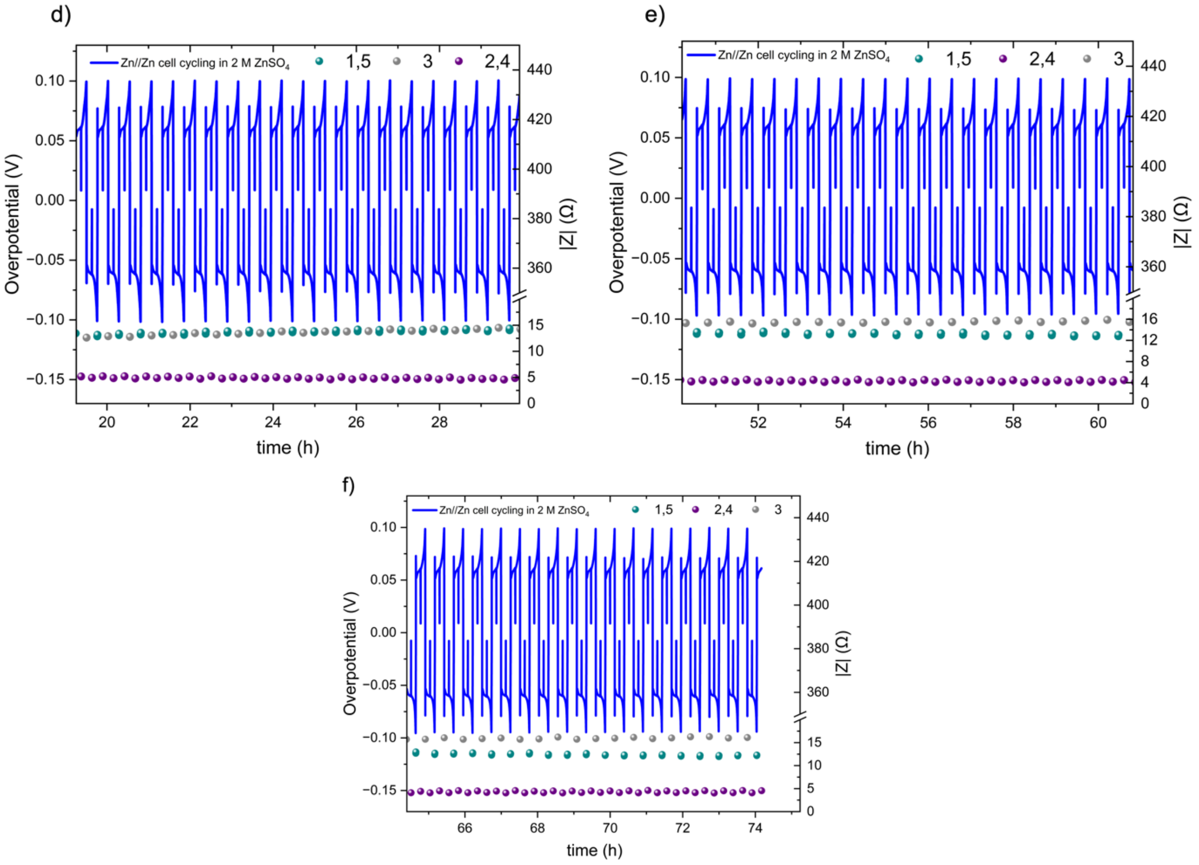

Figure S39: (a) An overpotential trace (overpotential vs t) of successive galvanostatic cycling of a total of 1.25 mAh cm-2 of Zn at 10 mA cm-2 in zinc symmetric cell with 2 M ZnSO4 aqueous electrolyte, GF separator, at 10 °C (left Y axe) and the total impedance at 10 Hz, measured during OCV (right Y axe). The galvanostatic impedance spectra (GEIS) were recorded every 1.25 hours, between 500 - 10 Hz with 100 µA amplitude. The single-spectrum measurement time was nine seconds. A drop in the overpotential (a spike in the overpotential trace) was observed when the GEIS measurements were taken at stages II and IV (b-f).

XPS Analysis

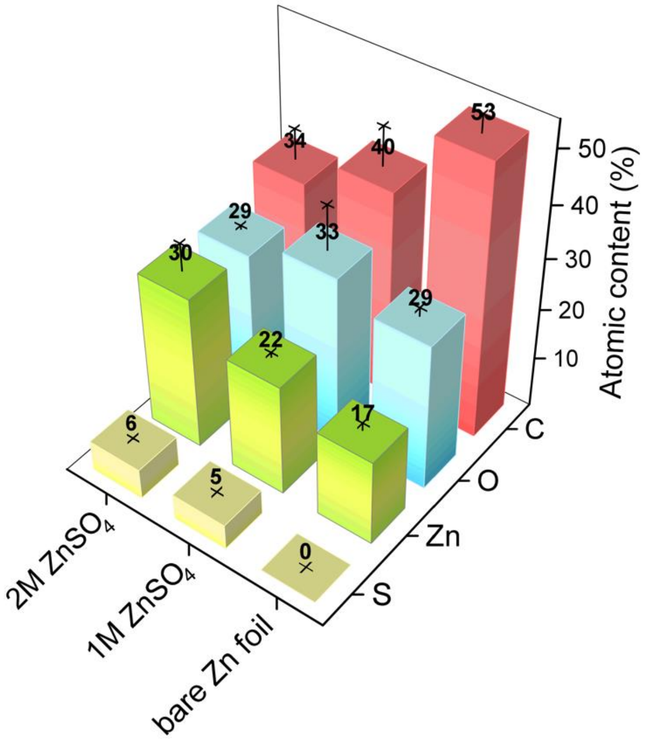

Figure S40: Atomic concentration bar graph. The atomic composition was derived from two points on the sample. The standard deviations are represented with lines and crosses. The presence of carbon is associated with a carbon coating of the Zn foil.

Table S1:Atomic content ratios in the XPS analysed samples.

**Sample/ atomic ratio**	**Zn/O**	**Zn/S**
**Bare Zn**	0.6	NA
**ZnSO4 theoretical**	0.25	1
**Zn4(OH)6SO4xH2O**	0.4	4
**Zn-SEI in 1M ZnSO4**	0.6	4.8
**Zn-SEI in 2M ZnSO4**	1	5.7

Raman Analysis

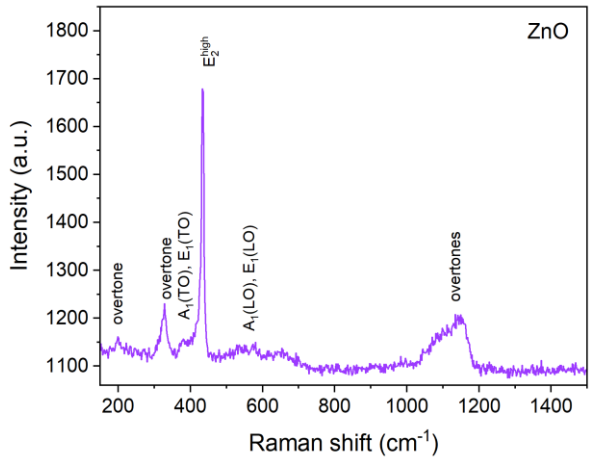

Figure S41: Reference Raman Spectrum for ZnO.

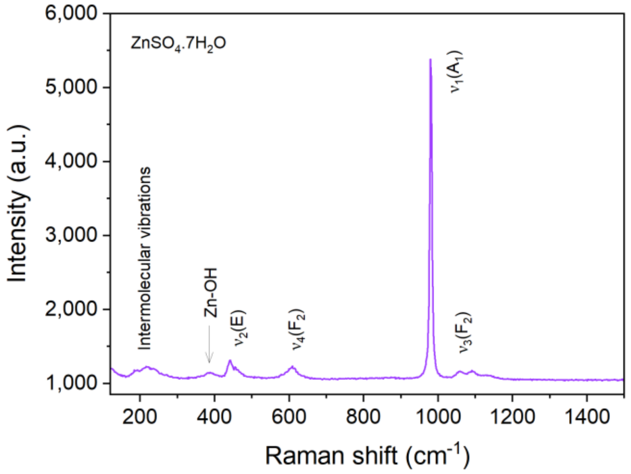

Figure S42: Reference Raman spectrum for $ZnSO_4.7H_2O$

Figures S43 and S44 show the Raman spectra for 1 M and 2 M $ZnSO_4$ solutions. The 1 M $ZnSO_4$ spectrum is red shifted compared to 2 M. The local potential field experienced by a particular ion varies in the presence of a counter ion field. As the concentration increases, the number of water molecules in the second solvation sphere changes, affecting the local potential field generated by the Zn^{2+} ion. Hence, the band maximum changes as the concentration of the solution changes. In this case, ions become more polarizable at high concentrations, shifting the band position to higher wavenumbers. Hence, the 1 M $ZnSO_4$ spectrum band is slightly red shifted compared to 2 M $ZnSO_4$.

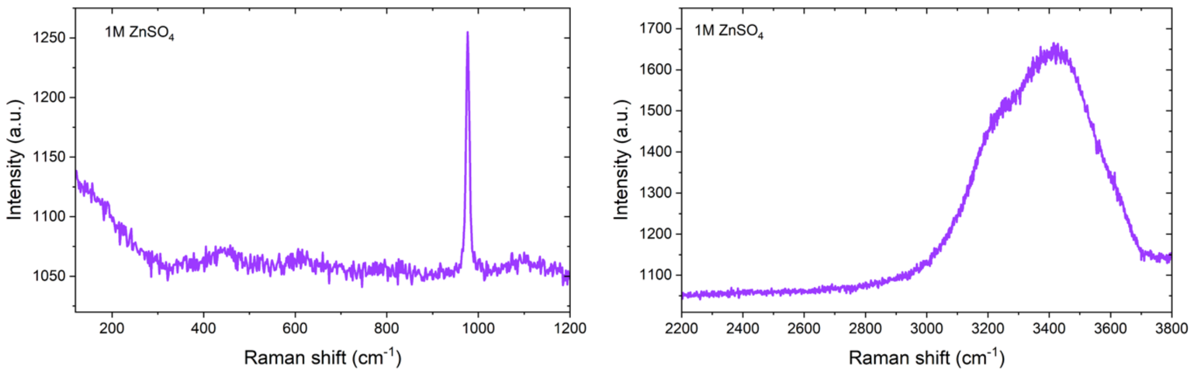

Figure S43: Reference Raman spectra for 1 M $ZnSO_4$ solution.

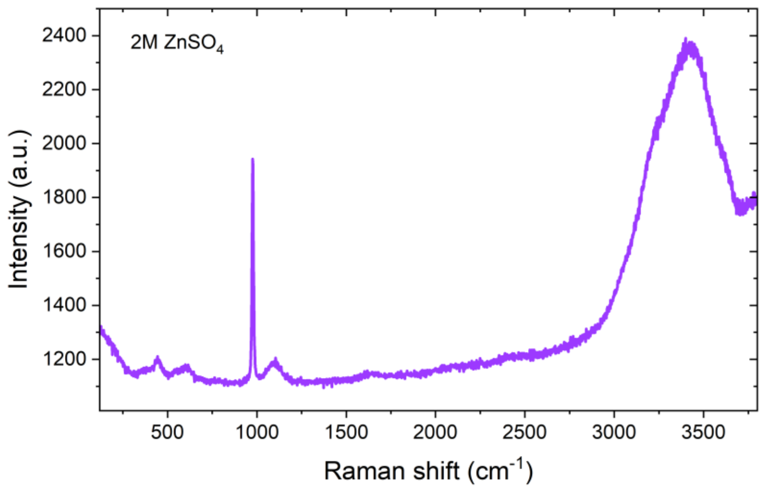

Figure S44: Reference Raman spectrum for 2 M $ZnSO_4$ solution.

The intensity of ν_1-SO_4^{2-} band for 2 M $ZnSO_4$ solution is higher compared to 1 M $ZnSO_4$ due to increased SO_4^{2-} concentration in 2 M solution. The relative intensity of this band increases with increasing the $ZnSO_4$ concentration in the solution.

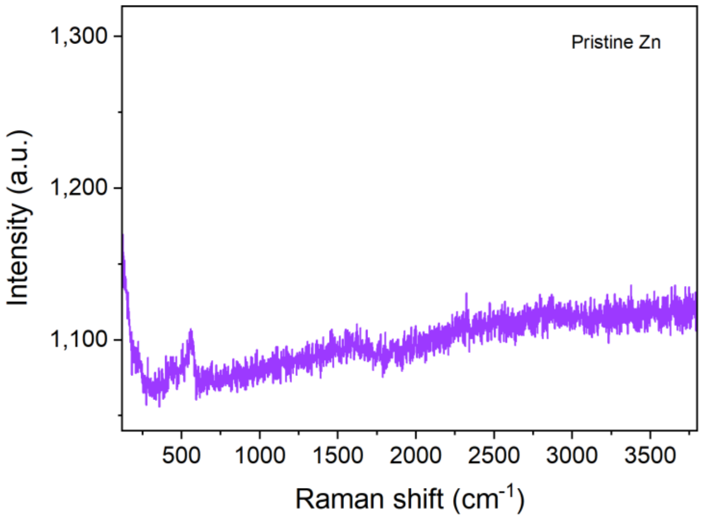

Figure S45: Reference spectrum for pristine zinc foil.

The presence of 565 cm^{-1} band in the spectrum indicates the presence of amorphous ZnO on the pristine Zn surface. However, the band is not observed at all the points which indicates the heterogeneous distribution of ZnO on the pristine Zn surface.

ZnO band does not show up in the Raman spectrum for the Zn cycled in 1 M $ZnSO_4$ solution while it is observed in 2 M $ZnSO_4$ solution. Keeping in mind the heterogeneity of ZnO distribution on pristine Zn surface, we believe the observed ZnO band in cycled Zn in 2 M $ZnSO_4$ solution comes from the pristine Zn surface.

SEM Images

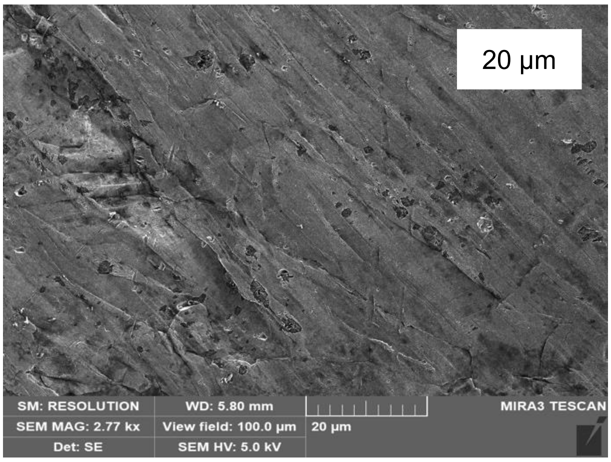

Figure S46: SEM Image of a zinc electrode soaked in 1 M $ZnSO_4$ for one hour with a view field of 100 µm view field. SEM image was taken with an excitation voltage of 5 kV in resolution mode.

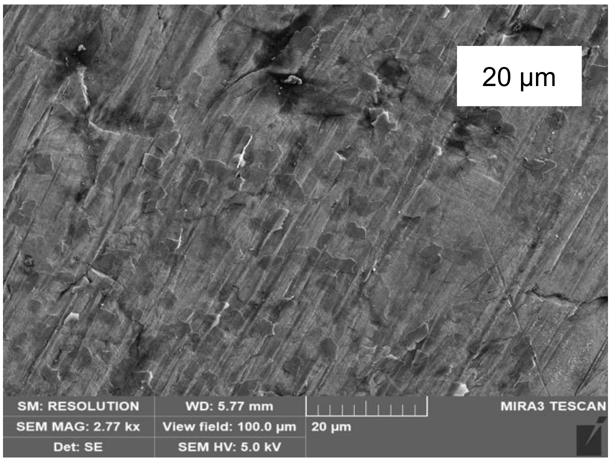

Figure S47: SEM Image of a zinc electrode soaked in 2 M $ZnSO_4$ for one hour with a view field of 100 µm view field. SEM image was taken with an excitation voltage of 5 kV in resolution mode.

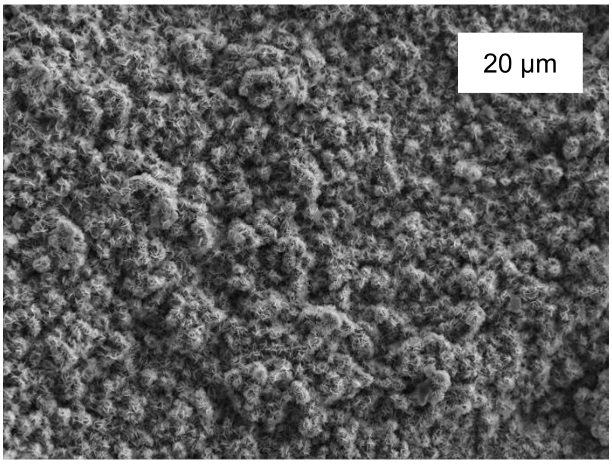

Figure S48: SEM Image after the first plating step in 2 M $ZnSO_4$ (view field 100 µm). SEM image was taken with an excitation voltage of 5 kV in resolution mode.

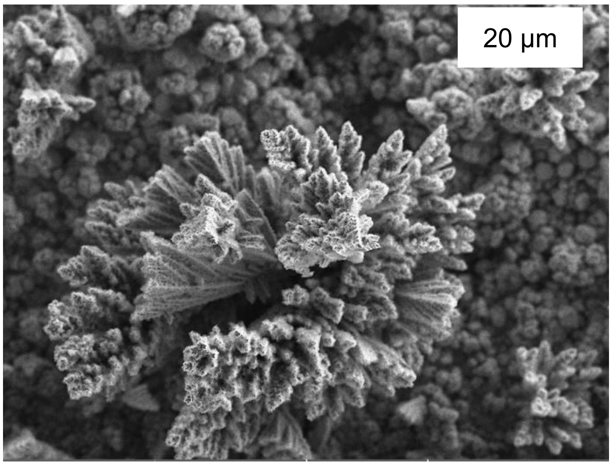

Figure S49: SEM image after fifth plating step in 2 M $ZnSO_4$ (100 µm view field). SEM image was taken with an excitation voltage of 5 kV in resolution mode.

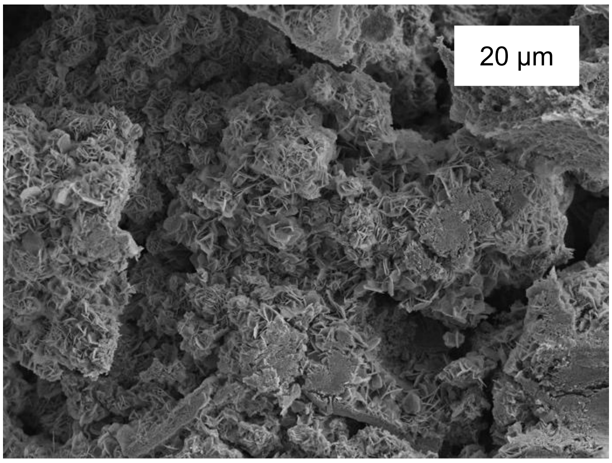

Figure S50: SEM image after tenth plating step in 2 M $ZnSO_4$ (100 µm view field). SEM image was taken with an excitation voltage of 5 kV in resolution mode.

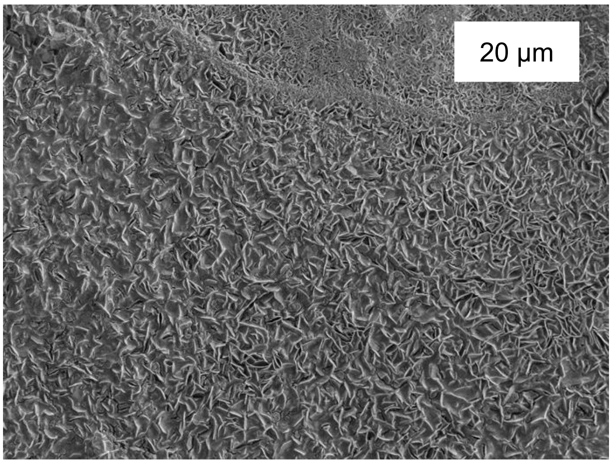

Figure S51: SEM image after first plating step in 1 M $ZnSO_4$ (100 µm view field). SEM image was taken with an excitation voltage of 5 kV in resolution mode.

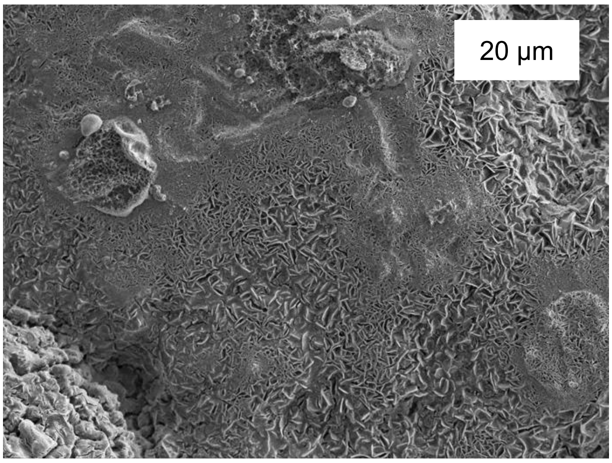

Figure S52: SEM image after fifth plating step in 1 M $ZnSO_4$ (100 µm view field). SEM image was taken with an excitation voltage of 5 kV in resolution mode.

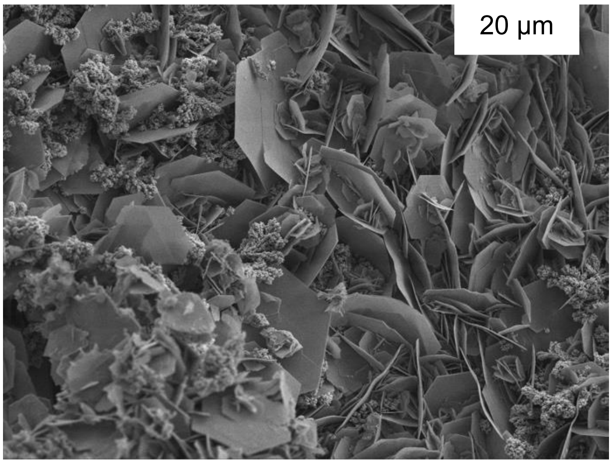

Figure S53: SEM image after tenth plating step in 1 M ZnSO4 (100 µm view field). SEM image was taken with an excitation voltage of 5 kV in resolution mode.

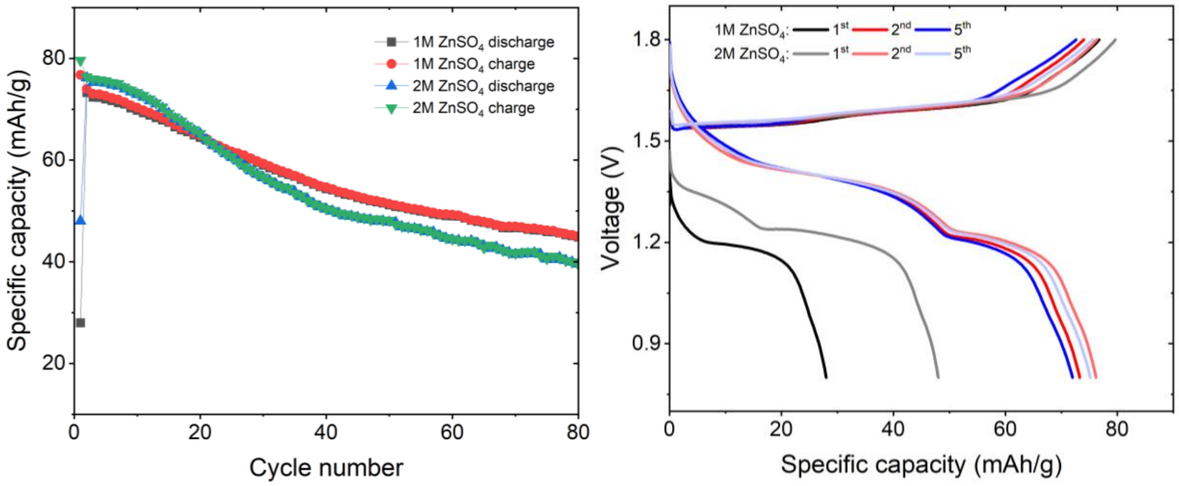

Figure S54: a) Cycling performance and (b) selected galvanostatic charge-discharge curves of Zn-Mn_2O_3 cells at 100 mA g^{-1} using 1 M and 2 M $ZnSO_4$ electrolytes.

Figure S54 depicts the cycling of Mn_2O_3 cathode vs. zinc metal. Since Mn_2O_3 requires activation before delivering a stable capacity, the Mn_2O_3 electrodes are initially cycled in the voltage range of 0.8-1.8 V for 15 cycles at 25 mA g^{-1}, and then the cell is cycled at a larger voltage range. Hence, the performance of the cell should be compared for the following cycles.

The cell with the 2 M electrolyte delivers a slightly higher capacity for initial cycles than the cell with the 1 M electrolyte. However, the capacity drops after 20 cycles, and 1 M $ZnSO_4$ electrolyte shows better capacity retention at the end of 80 cycles. The voltage profiles are similar in both the electrolytes except for the first cycle after activation.

References

1 A. S. Cuharuc, G. Zhang and P. R. Unwin, *Phys. Chem. Chem. Phys.*, 2016, **18**, 4966–4977.
2 S. Ching, R. Dudek and E. Tabet, *J. Chem. Educ.*, 1994, **71**, 602.
